# ‘Spikeopathy’: COVID-19 Spike Protein Is Pathogenic, from Both Virus and Vaccine mRNA

**DOI:** 10.3390/biomedicines11082287

**Published:** 2023-08-17

**Authors:** Peter I. Parry, Astrid Lefringhausen, Conny Turni, Christopher J. Neil, Robyn Cosford, Nicholas J. Hudson, Julian Gillespie

**Affiliations:** 1 Children’s Health Research Clinical Unit, Faculty of Medicine, The University of Queensland, South Brisbane, QLD 4101, Australia; 2 Department of Psychiatry, College of Medicine and Public Health, Flinders University, Bedford Park, SA 5042, Australia; 3 Children’s Health Defence (Australia Chapter), Huskisson, NSW 2540, Australia; alefringhausen@gmail.com (A.L.); robyncosford@gmail.com (R.C.); juliangillespie69@gmail.com (J.G.); 4 Microbiology Research, QAAFI (Queensland Alliance for Agriculture and Food Innovation), The University of Queensland, St. Lucia, QLD 4072, Australia; c.turni1@uq.edu.au; 5 Department of Medicine, University of Melbourne, Melbourne, VIC 3010, Australia; christopher.neil@heartweb.com; 6 School of Agriculture and Food Science, The University of Queensland, Brisbane, QLD 4072, Australia; n.hudson@uq.edu.au

**Keywords:** spike protein, pathology, transfection, biodistribution, lipid-nanoparticles, autopsy, inflammation, pharmacovigilance, COVID-19, mRNA vaccines

## Abstract

The COVID-19 pandemic caused much illness, many deaths, and profound disruption to society. The production of ‘safe and effective’ vaccines was a key public health target. Sadly, unprecedented high rates of adverse events have overshadowed the benefits. This two-part narrative review presents evidence for the widespread harms of novel product COVID-19 mRNA and adenovectorDNA vaccines and is novel in attempting to provide a thorough overview of harms arising from the new technology in vaccines that relied on human cells producing a foreign antigen that has evidence of pathogenicity. This first paper explores peer-reviewed data counter to the ‘safe and effective’ narrative attached to these new technologies. Spike protein pathogenicity, termed ‘spikeopathy’, whether from the SARS-CoV-2 virus or produced by vaccine gene codes, akin to a ‘synthetic virus’, is increasingly understood in terms of molecular biology and pathophysiology. Pharmacokinetic transfection through body tissues distant from the injection site by lipid-nanoparticles or viral-vector carriers means that ‘spikeopathy’ can affect many organs. The inflammatory properties of the nanoparticles used to ferry mRNA; N1-methylpseudouridine employed to prolong synthetic mRNA function; the widespread biodistribution of the mRNA and DNA codes and translated spike proteins, and autoimmunity via human production of foreign proteins, contribute to harmful effects. This paper reviews autoimmune, cardiovascular, neurological, potential oncological effects, and autopsy evidence for spikeopathy. With many gene-based therapeutic technologies planned, a re-evaluation is necessary and timely.

## 1. Introduction

In this narrative review, we examine the solid evidence for a counter-narrative to the ‘safe and effective’ message that has accompanied the novel product COVID-19 vaccines, which were developed at ‘warp speed’ with great hope to end the pandemic. This evidence has accumulated and dampened the original optimism. The implications for the recognition of vaccine-related diagnoses and the need for therapeutics are significant for all health practitioners and many research scientists to consider.

Key problem areas appear to be (1) the toxicity of the spike protein—both from the virus and also when produced by gene codes in the novel COVID-19 mRNA and adenovectorDNA vaccines [1,2], hence the novel term ‘spikeopathy’; (2) inflammatory properties of certain lipid-nanoparticles used to ferry mRNA [3]; (3) N1-methylpseudouridine in the synthetic mRNA that causes long-lasting action [4]; (4) widespread biodistribution of the mRNA [5] and DNA [6,7] codes via the lipid-nanoparticle and the viral-vector carrier matrices, respectively and (5) the problem of human cells producing a foreign protein in our ribosomes that can engender autoimmunity [8,9].

The emergence of SARS-CoV-2 in late 2019, and the associated disease of COVID-19, declared by March 2020 as a global pandemic by the WHO, has caused much illness, and many deaths in the elderly and the at-risk, and seriously disrupted society. An umbrella literature review of publications between December 2019 and August 2021 revealed that the greatest risk of mortality due to COVID-19 was associated with cardiovascular disease, cerebrovascular disease, and chronic renal disease [10]. The production of safe and effective vaccines to halt the COVID-19 pandemic was one of the most important public health interventions. Many COVID-19 vaccines have been developed across the world. In non-Western nations, most vaccines have used traditional protein-based or inactivated virus technologies. The mRNA and adenovectorDNA vaccines have been produced by large pharmaceutical companies and favoured by regulators in most Western nations. It has been widely claimed that these vaccines have saved millions of lives. Sincere hopes have been held for this narrative. But this belief is largely founded on early Infection Fatality Rate (IFR) modelling estimates and Pfizer, Moderna, AstraZeneca and Janssen claims of efficacy, which have been undermined by new data.

Controversy has surrounded the use of the gene-based vaccines and this article explores the reason for this. To meet the widespread desire for ‘safe and effective’ vaccines, gene-based technology offers rapid speed of production. Hope has perhaps influenced much of the published literature as well as media narrative. A central issue has been growing evidence of pathogenic effects of the SARS-CoV-2 spike protein—whether as part of the virus or produced by genetic codes in the mRNA and adenovectorDNA vaccines.

The aim of this narrative review is to present a comprehensive account of the pathogenicity of the antigen, the biodistribution of the gene codes for the antigen throughout the body, their modified long-lasting nature particularly with the mRNA vaccines, and literature and data that show the adverse events that would be expected from such biodistribution and cellular production of a foreign antigen. The review presents a case of premature translation of experimental gene therapy technology to mass public vaccination and ethical and regulatory issues that need scrutiny and reform before the next pandemic. 

Central to individual informed consent decisions and public health policy is the weighing of the risks of an illness versus the risks and potential benefits of an intervention. Given the risks of novel gene-based COVID-19 vaccines, were they worth it in light of the severity of SARS-CoV-2 infection? We address the risks of COVID-19 first.

## 2. COVID-19 Modelling Versus Real-World Data

It is apparent that the original Wuhan strain and early variants of SARS-CoV-2 in 2020 were more pathogenic than later variants. This is consistent with typical viral adaptive evolution to more infectious but less pathogenic strains, a natural phenomenon that is fortunate for humanity. The claim that the COVID-19 vaccines have saved many millions of lives is predicated on modelling based on case fatality rates (CFR) in China in February 2020 published by Verity et al. in *The Lancet* [11]. The authors estimated a CFR of 6.4% (5.7–7.2) in those aged over 60 years and “up to 13.4% (11.2–15.9) in those aged 80 years or older… with an overall infection fatality ratio for China of 0.66% (0.39–1.33)” (abstract). Fortunately, the virus mutated, and these modelling predictions did not materialise as the pandemic unfolded over the next three years.

The COVID-19 vaccines have saved lives from COVID-19, but it is not clear how many. The claim of millions of lives saved by COVID-19 gene-based vaccines was partly based on assumptions that the COVID-19 vaccines protected against infection and transmission, which was not the case because systemic immunity to respiratory viruses is not as effective as mucosal immunity from infection, and because of the continually evolving variants perhaps partly driven by adaptive evasion of vaccine-induced antibodies. Pfizer admitted that its phase 3 clinical trial [12] did not test for viral transmission [13]. 

However, presumptions of efficacy have been sustained by COVID-19 modellers, and reiterated by health authorities, medical publications, and the media. This is exhibited by Watson et al., (2022) in “Global impact of the first year of COVID-19 vaccination: a mathematical modelling study”, published in *The Lancet Infectious Diseases* [14]. The authors estimate around 14.4 million lives saved related to vaccination benefits that include protection against infection and transmission, both now recognised to be unfounded. This suppositional estimate by Watson et al. persists as an accepted fact, whereas real-world infection fatality rate (IFR) data speak against the need for vaccination in the non-elderly. 

Briefly, Roussel et al. in early 2020 presented a statistically significant analysis that likened the case fatality rate for SARS-CoV-2 to earlier coronaviruses and influenza-like illnesses: In OECD countries, the mortality rate for SARS-CoV-2 (1.3%) was not significantly different from that for common coronaviruses identified in public hospitals of Marseille, France (0.8%; *p* = 0.11) [15]. If modelling had been based on these data a few months after the initial Chinese data, different projections would have been made, more in line with eventual mortality statistics including in 2020 prior to any vaccine availability.

Ioannidis et al. in 2022 in a paper titled “Forecasting for COVID-19 has failed” critiqued the models that ignored the low IFRs to emerge in the first half of 2020 [16]. Ioannidis et al. noted:

“*Failure in epidemic forecasting is an old problem. In fact, it is surprising that epidemic forecasting has retained much credibility among decision-makers, given its dubious track record. Modelling for swine flu predicted 3100–65,000 deaths in the UK (https://www.theguardian.com/uk/2009/jul/16/swine-flu-cases-rise-britain. (Accessed on 2 June 2020). Eventually, 457 deaths occurred (UK government, 2009)*”.

[16] (p. 425)

Ioannidis et al. then examined many US COVID-19 prediction models for deaths, hospitalisations, and ICU admissions, highlighting the extremely wide margins by which they failed to hit their targets. Ioannidis et al. continued:

“*Despite these obvious failures, [COVID-19] epidemic forecasting continued to thrive, perhaps because vastly erroneous predictions typically lacked serious consequences… Upon acquiring solid evidence about the epidemiological features of new outbreaks, implausible, exaggerated forecasts (Ioannidis, 2020d) should be abandoned. Otherwise, they may cause more harm than the virus itself*”.

[16] (p. 428)

Societal narratives, once entrenched, become difficult to shift.

Accurate estimates of lives saved or lost as a result of the COVID-19 gene-based vaccines would have required long-term studies in vaccinated compared to unvaccinated individuals. Pfizer, Moderna, AstraZeneca and Janssen eventually vaccinated almost all placebo subjects and thus lost their control group. This was based on ethical principles given the fear of COVID-19 [17], but the loss to scientific integrity of only having short-term placebo-controlled trials was noted by the WHO Ad Hoc Expert Group on the Next Steps for Covid-19 Evaluation (2020) [18]. 

To make up this deficit, one private organisation based in the UK, Control Group Cooperative [19], has collected data since the COVID-19 vaccination rollout, and is the only world-wide control group. Of this unvaccinated cohort 18,497 participated in a survey reporting COVID-19 positive testing and symptom severity between September 2021 and February 2022. A quarter (4636, 25.1%) reported experiencing symptomatic COVID-19 illness. Symptoms were reported as “mild” by 14.4%, “moderate” by 8.7% and “severe” by 2%. A further 560 reported asymptomatic illness and of the 5196 with COVID-19, only 74 (1.4%) reported attending hospital (as in- or out-patients) with 21 (0.4%) being hospitalised for longer than 1 week. As a self-reported survey, the limitations included deaths that may not have been reported; nonetheless, the cohort fared better than expected. The group was perhaps unusual in that 71% partook of some combination of vitamins C, D, quercetin, zinc and off-label ivermectin or hydroxychloroquine where available [20].

**Figure 1:**
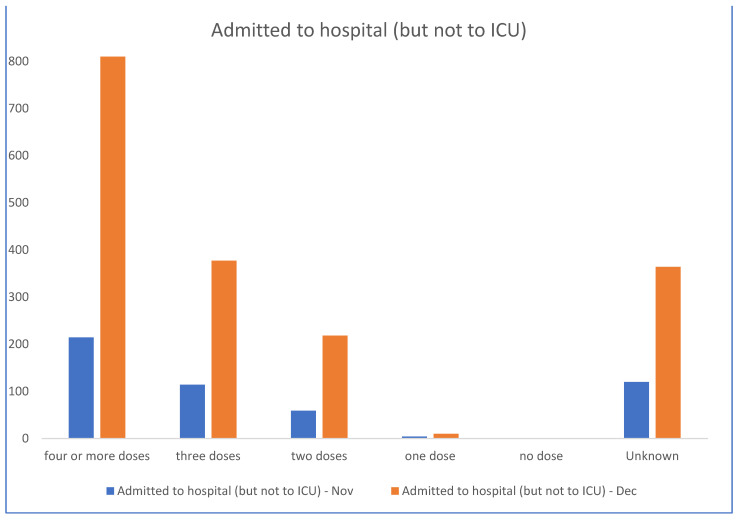

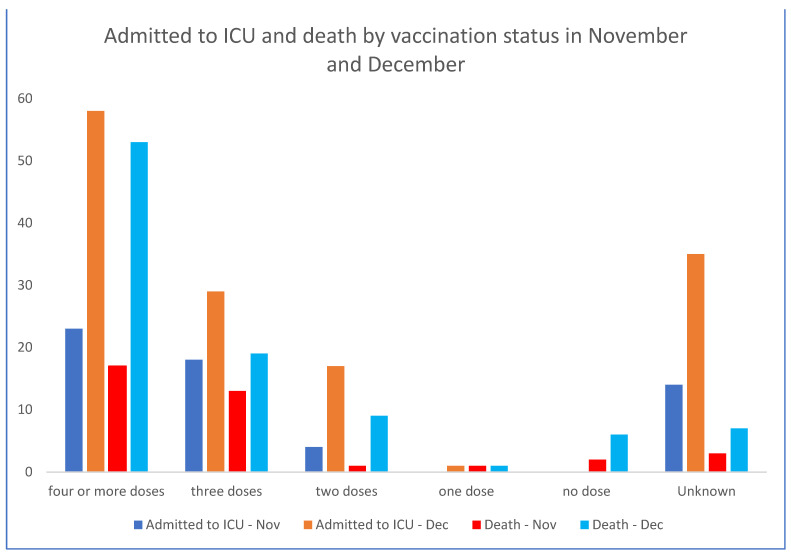
NSW Australia hospitalisations, ICU admissions and deaths last 6 weeks 2022 by vaccination status. NSW Health. Bar charts derived from the numbers in official government report excerpt of posted as Figure 2 [21].

**Figure 2:**
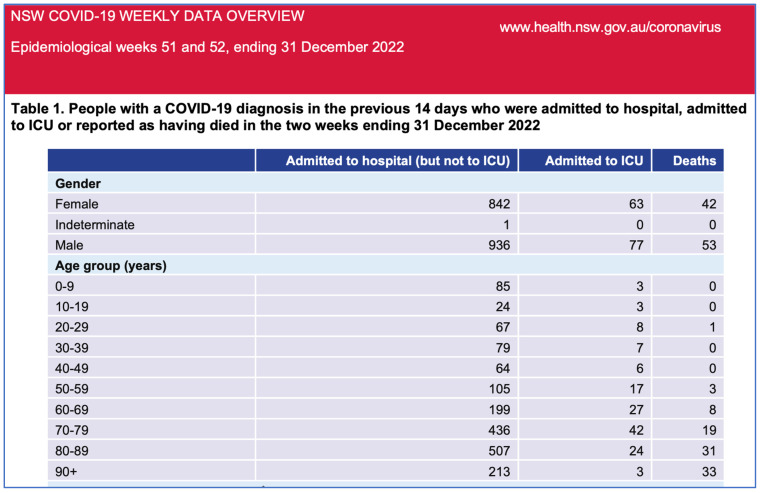

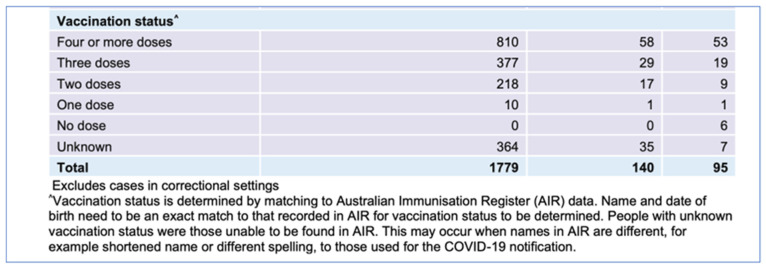
NSW Australia COVID-19 hospitalisations, ICU admissions, deaths, last 2 weeks 2022. NSW Health. From Table 1 of NSW Covid weekly data overview last 2 weeks 2022. Note that regional councils analysis of same data removed for space reasons. Used under Creative Commons Attribution 4.0 license. © State of New South Wales. For current information go to www.nsw.gov.au. [21].

In this context, the Australian State Government (NSW) health data from November and December 2022 [21] (Figure 1 and Figure 2) demonstrate that the unvaccinated are almost not represented in the hospitalisation data while the most vaccinated are over-represented. The proportion of unvaccinated in NSW was low at 3.2%; however, the proportion of unvaccinated with severe COVID-19 is lower than this in late 2022 at 2.9%. Even accounting for more COVID-19 vaccine boosters in the elderly and vulnerable, the data do not suggest significant efficacy against hospitalisation, ICU admission and death, at least after the emergence of the Omicron strain.

For weeks 51 and 52 of 2022, the NSW government data document nil hospitalisations and six deaths for unvaccinated persons, but 1415 hospitalisations and 82 deaths in known vaccinated persons. NSW Health no longer publishes vaccination status. These data do not support the premise that the vaccinations have ‘saved millions of lives’, but instead indicate correlations between more doses with severe COVID-19 illness warrants investigation. There has been an increase in all-cause mortality contemporaneous with the rollout of the COVID-19 gene-based vaccines and this warrants further research.

Mathematical models produce highly uncertain numbers that predict the future. These predictions can become politicised. To make sure predictions do not become adjuncts to a political cause, modellers, decision-makers and citizens need to establish the real-world facts that hold us all accountable. 

If the COVID-19 vaccines are less efficacious than was originally hoped for and subsequently claimed, then the risk/benefit decision-making for individual informed consent and public health policy shifts. The degree of harm caused by the novel gene-based vaccine technology might then outweigh any benefits.

## 3. Correspondence between TGA and Australian Senator Rennick

In Australia, the Therapeutic Goods Administration (TGA) provisionally approved the COVID-19 vaccines of Pfizer (Comirnaty, BNT162b2), Moderna (SPIKEVAX, mRNA-1273), AstraZeneca (Vaxzevria, ChAdOx1 nCOV-19) and Janssen (COVID-19 Vaccine, Ad26.COV2.S) in early 2021 [22] and in January 2022 added the protein-based lipid-nanoparticle embedded vaccine of Novavax (Nuvaxovid, NVX-CoV2373) [23].

On 16 December 2022, the Australian Department of Health advised by the TGA responded to Question 235 from 21 November 2022 by Senator Gerard Rennick (Liberal Party, Qld) in the Senate Community Affairs Committee Question on Notice SQ22-000609. Senator Rennick, whose parliamentary office has received numerous accounts of COVID-19 vaccine injuries from Australians, had asked whether the TGA’s own report [5] that showed widely biodistributed high transfection and expression rates of the gene-based COVID-19 mRNA vaccines, was proof the vaccines were more pathogenic than the virus, implying more spike protein load on human cells [24].

The TGA replied:

“*There is some confusion around the biochemistry and immunology here. Higher translation and expression rate is not associated with pathogenicity, rather it indicates better antigen (spike protein) expression. The expressed spike protein is not a pathogen and is not infectious. The spike protein is only one component of the coronavirus. It serves as an antigen to induce humoral and cellular immune responses against SARS-CoV-2 virus*”.

[24]

As Australian authors of this paper, we concur with the opinion of the TGA that the spike protein produced by the gene-based COVID-19 vaccines does act as an antigen to induce immune responses and is not a whole microorganism pathogen. However, the response by the TGA has missed the point of the question. We will summarise the evidence that the spike protein itself is independently bioactive and pathogenic. The spike protein has been directly related to both the pathophysiology that underlies COVID-19 viral illness and the serious adverse events from the COVID-19 vaccines that, via gene therapy mechanisms, induce human cells to produce the spike protein in substantial numbers. 

In fact, the spike protein in the original SARS coronavirus 1 (SARS CoV-1) epidemic in 2003 was identified as a cause of lung injury for which the term ‘severe acute respiratory syndrome’ (SARS) was coined. It was thought to do this via action on angiotensin-converting enzyme 2 (ACE-2) receptors. SARS-CoV-1 (2003 virus) spike protein-driven downregulation of ACE-2 receptors led to lung oedema and acute pulmonary failure in mice as published in *Nature Medicine* [25]. 

## 4. Narrative Review Methodology

We present here a narrative review of the literature that provides evidence for the toxicity and thus pathogenicity of the spike protein, independent of its role as a pathogenic determinant in SARS-CoV-2 infection. This is whether from the SARS-CoV-2 virus or produced by genetic code in human cells directly by mRNA (Pfizer and Moderna) or by mRNA derived from the adenovectorDNA (AstraZeneca and Janssen) COVID-19 vaccines. 

We also review literature evidence for the toxicity and biodistribution profile of concern for the lipid-nanoparticle matrices for mRNA Moderna and Pfizer and protein-based Novavax COVID-19 vaccines; the modified nature of the synthetic mRNA which would explain prolonged mRNA persistence and spike protein production; the phenomenon of ‘bad batch’ variation in adverse event reports and relevant age-stratified risk/benefit considerations for COVID-19 vaccinations especially for paediatric and younger adult age cohorts. 

These pharmacokinetic and pharmacodynamic aspects relate to the pathogenicity of the gene-based COVID-19 vaccines. In the context of the TGA’s reply above, the pharmacokinetic biodistribution aspects of the gene-based COVID-19 vaccines are akin to an ‘infectious’ agent, in an invasive or blood-borne phase, as they distribute the pathogenic effects of the spike protein throughout the body. 

This review presents evidence from the academic literature, as well as pharmacovigilance, and Pfizer clinical trial documents, via Freedom of Information (FOI) orders, to assist the TGA and other regulators and health authorities in reappraising the toxicity of the mRNA and adenovectorDNA produced spike proteins. A new era of pathology is emerging that could be termed “Spikeopathy”. It is also vital to evaluate the potential for any new autoimmune phenomena driven by foreign antigen production caused by any new mRNA or DNA-based technology in the future. 

Evidence for harm caused by ‘spikeopathy’, as well as other forms of pathophysiological damage, are reviewed by organ system, while a review of pharmacovigilance data will be the subject of a further paper. 

The Key Points below summarise the information presented.

### Key Points

- Highly safe and effective vaccines are central to combat infectious disease epidemics/pandemics.
- SARS-CoV-2 spike protein is pathogenic, whether from the virus or created from genetic code in mRNA and adenovectorDNA vaccines.
- Biodistribution rodent study data show lipid nanoparticles carry mRNA to all organs and cross blood-brain and blood-placenta barriers. Some of these tissues are likely to be impervious to viral infection; therefore, the biohazard is particularly from vaccination.
- Lipid-nanoparticles have inflammatory properties.
- The modification of mRNA with N1-methylpseudouridine for increased stability leads to the production of spike proteins for months. It is uncertain how many cells and from which organs mRNA spike proteins are produced, and therefore, the exact effective dose delivered per vaccine vial is unknown.
- The long-term fate of mRNA within cells is currently unknown.
- The mRNA and adenovectorDNA vaccines act as ‘synthetic viruses’.
- In the young and healthy, and even in many older individuals with vulnerable comorbidities, the encoding-based COVID-19 vaccines will likely transfect a far more diverse set of tissues than infection by the virus itself.
- Evidence suggests reverse transcription of mRNA into a DNA copy is possible. This further suggests the possibility of intergenerational transmission if germline cells incorporate the DNA copy into the host genome.
- Production of foreign proteins such as spike protein on cell surfaces can induce autoimmune responses and tissue damage. This has profoundly negative implications for any future mRNA-based drug or vaccine.
- The spike protein exerts its pathophysiological effects (‘spikeopathy’) via several mechanisms that lead to inflammation, thrombogenesis, and endotheliitis-related tissue damage and prion-related dysregulation.
- Interaction of the vaccine-encoded spike protein with ACE-2, P53 and BRCA1 suggests a wide range of possible biological interference with oncological potential.
- Adverse event data from official pharmacovigilance databases, an FDA-Pfizer report obtained via FOI, show high rates and multiple organ systems affected: primarily neurological, cardiovascular, and reproductive.
- Pfizer and Moderna mRNA COVID-19 vaccines’ clinical trial data independently interpreted has been peer-review and published to show an unfavourable risk/benefit, especially in the non-elderly. The risks for children clearly outweigh the benefits.
- Repeated COVID-19 vaccine booster doses appear to induce tolerance and may contribute to recurrent COVID-19 infection and ‘long COVID’.
- The SARS-CoV-2 pandemic has revealed deficiencies in public health and medicines regulatory agencies.
- A root cause analysis is needed for what now appears a rushed response to an alarming infectious disease pandemic.
- Treatment modalities for ‘spikeopathy’-related pathology in many organ systems, require urgent research and provision to millions of sufferers of long-term COVID-19 vaccine injuries.

## 5. Structure of SARS-CoV-2 Spike Protein

**Figure 3:**
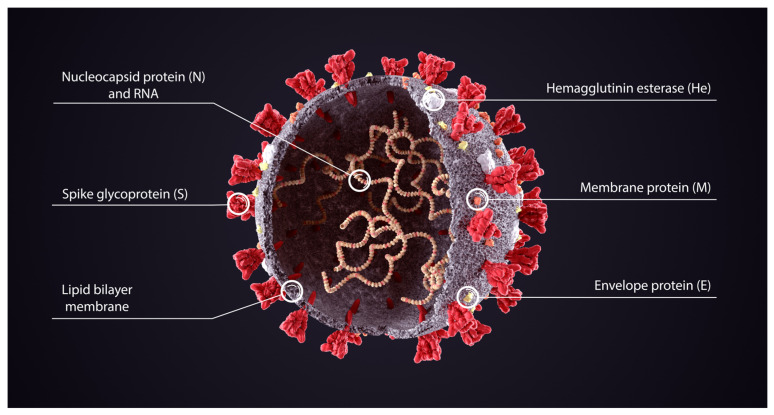
Diagram of various proteins of SARS-CoV-2 virus. Reprinted from *News-Medical.net* (accessed on 26 April 2023) Cuffari (2021): What are spike proteins? (*with permission, license from Shuttercock*). [27].

Cryo-EM electron microscopy revealed the structure of the spike protein at the outset of the pandemic [26]. The SARS-CoV-2 spike proteins protrude outwards from the cell wall of the virus and are in red in the schematic diagram in Figure 3 from Cuffari [27]. 

In the context of SARS-CoV-2 infection, the spike protein is a pathogenic determinant of cell invasion, consisting of two subunits: S1 at the distal end of the spike glycoprotein pointing outwards from the virus constructed of an N-terminal domain (NTD) and a trimer of three receptor binding domains (RBD), and S2 consisting primarily of a C-terminal region that forms the stalk of the spike protein and embeds proximally to the virus’ envelope or membrane. 

The virus uses the spike protein to bind with ACE-2 receptors on cell surfaces to enter the cells. For this to happen, the receptor binding domain (RBD) on the S1 subunit undergoes hinge-like extension from the ‘down’ to ‘up’ position to interact with the ACE-2 receptor.

**Figure 4:**
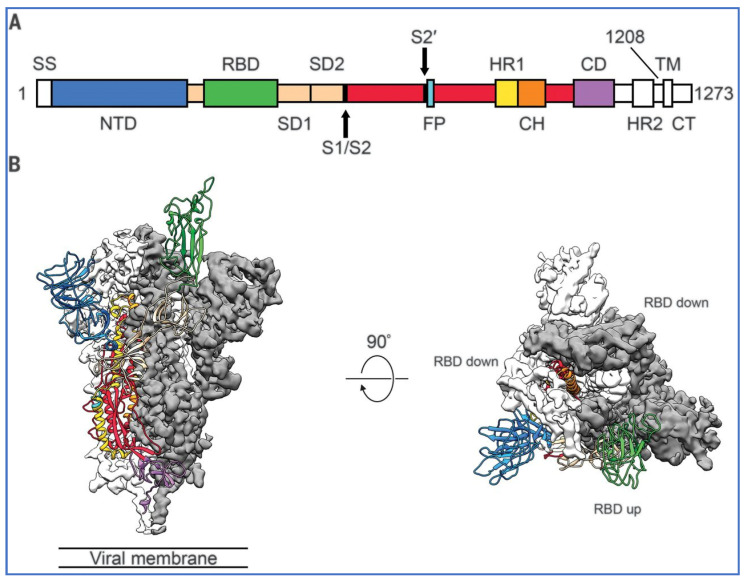
Structure of 2019-nCoV S in the prefusion conformation. (**A**) Schematic of 2019-nCoV S primary structure coloured by domain. Domains that were excluded from the ectodomain expression construct or could not be visualised in the final map are coloured white. SS, signal sequence; S2′, S2′ protease cleavage site; FP, fusion peptide; HR1, heptad repeat 1; CH, central helix; CD, connector domain; HR2, heptad repeat 2; TM, transmembrane domain; CT, cytoplasmic tail. Arrows denote protease cleavage sites. (**B**) Side and top views of the prefusion structure of the 2019-nCoV S protein with a single RBD in the up conformation. The two RBD down protomers are shown as cryo-EM density in either white or gray and the RBD up protomer is shown in ribbons coloured corresponding to the schematic in (**A**). Reprinted from [26] Figure 1, Copyright (2022) *with permission*.

Figure 4, from Wrapp et al. [26], shows one of the three ‘trimer’ RBDs in green in the ‘up’ position while the other two RBDs are ‘down’ and inaccessible to the attachment to ACE-2. The diagram on the left is the view of the spike protein in profile and on the right is a view of the S1 subunit or top of the trimeric spike protein from above. 

### 5.1. Does the Vaccine Produced Spike Protein Have Protective Closed RBDs?

The SARS-CoV-2 virion carries spike protein in the form of trimers, predominantly in prefusion form. Prefusion spike protein trimers on each virus are found in various conformations, either closed with all three RBDs lying down at the top of the spike—or open, in which one or more of the RBDs protrude from the top of the spike. The receptor binding site (RBS) is largely inaccessible when the RBDs are in the down position. Spike protein contains a furin cleavage site, where it can be split into S1 and S2 subunits which facilitates infectivity. Serine protease is necessary to split the spike protein into S1 and S2 subunits which greatly increases infectivity via the ACE-2 receptor. 

After interaction with the receptor, the spike protein undergoes a conformational rearrangement leading to exposure of the S2 subunit, insertion of the fusion peptide into the membrane of the target cell, and refolding of S2. This refolding pulls the fusion peptide and transmembrane domain of the spike protein together, drawing the target cell and viral membranes together and causing their fusion. As an analogy, imagine a bottle opener pulling the cork up from the bottle neck—but the cork is connected to a cell membrane that gets pulled up along with it [28].

The commercially available vaccines in Australia rely on engineered mutations in the spike protein designed to stabilise the prefusion state and reduce the transition into the post-fusion form and therefore limit cleavage. Mutations include the replacement of two residues with a double proline (e.g., Pfizer/BioNTech, Moderna, Novavax, and Janssen), or mutations in the furin cleavage site for protease resistance (Janssen). 

Given amassed data that suggest mRNA and adenovectorDNA-created spike proteins cause harm, these theoretical safeguards appear to have failed. 

There are several possible reasons for the failure of this system. Since only the mRNA, not the full-length spike protein, gets injected with the lipid-nanoparticles, there is the possibility that the mRNA fragments are not full-length, due to suboptimal synthesis or degradation after manufacture. Spike protein could then be partially expressed as truncated spike protein with a conformation that allows cleavage into a peptide part and a functional S1 or S2 subunit. 

Even with full protein code expression, some cleavage can still happen inside cells. No biological system is 100% effective, and the mutation is only supposed to reduce, not completely prevent cleavage into S1 and S2. The transport of spike proteins or subunits via exosomes, direct cell fusion and nanotube tunnels to other cells is still possible. Expression errors inside the cell could lead to spike proteins retaining certain functions. Contamination with replication-capable plasmid vectors leaves the option of mutation during replication or insertion into the genome. 

The spike protein is not only toxic through binding of ACE-2 receptors, but it also has cytotoxic effects inside cells through interaction with cancer suppressor genes BRCA and P53 and mitochondrial damage, coagulopathies through direct contact with cellular proteins, and is neurotoxic through accumulation, with spread and reconfiguration of prion proteins into their pathologic form. The accumulation of spike protein inside cells could have toxic and apoptotic effects [29]. 

### 5.2. Toxin-Like Domain in the RBD

Another mechanism for pathogenicity has recently been demonstrated. The spike protein has been shown to also contain a ‘toxin- like’ domain in the RBD on S1, with sequence homology to Rabies Virus (RBG) and HIV glycoproteins, and neurotoxin NL-1, all of which bind to the α7 Nicotinic Acid Acetylcholine Receptors (α7 nAChR) of the cholinergic system [30]. Neurotoxin NL-1 is a neurotoxin, a type of snake venom, and similar to the archetypal bungarotoxin, a known inhibitor of the α7 nAChR, with high binding affinity. Snake venom three-finger neurotoxins (α-3FNTx) act on postsynaptic nicotinic acetylcholine receptors (nAChRs) at the neuromuscular junction (NMJ) to produce skeletal muscle paralysis and at specific nACHR at other sites [31], resulting in disturbances in the control of inflammation [32]. 

This spike toxin-like binding domain is a part of the RBD, adjacent to the ACE receptor binding site and has been demonstrated both in a computer-simulated study [32] and in electrophysiological studies, to bind preferentially to the α7 nAChR in nanomolar doses, similar to neurotoxins, such as bungarotoxin. The active peptide SCoV2P potentiates and inhibits acetylcholine (ACh)-induced α7 nAChR responses by a potential allosteric mechanism in nanomolar potencies and nicotine enhances these effects. At low doses, it potentiates and at higher doses, it inhibits nAChR function [33]. 

This binding model could provide logical explanations for the acute inflammatory disorder and other conditions in patients with COVID-19, long COVID, and vaccination injury, which may be linked to severe dysregulation of the central nervous system.

## 6. Reasons for Concern: Pharmacodynamic, Pharmacokinetic, and Pathophysiological

Pharmacokinetic and pharmacodynamic data give cause for concern about the conceptual design of the mRNA and adenovectorDNA COVID-19 vaccines and lay the groundwork for understanding the pathophysiology that is now being widely reported. There is uncontrolled biodistribution as well as durability and persistent bioavailability of the spike protein.

### 6.1. Gene-Based Vaccines Are Novel Experimental Technology

The unprecedented number of adverse events appears to be associated with the spike proteins produced by the gene-based technologies employed by Pfizer, Moderna, AstraZeneca, and Johnson and Johnson. Viral-vectorDNA technology is also employed in the Sputnik V and EpiVacCorona COVID-19 vaccines in Russia, iNCOVACC in India, and Convidecia in China. But the majority of COVID-19 vaccines, mostly made in non-Western countries, are traditional protein-based or inactivated virus non-genetic vaccines [34,35]. 

The gene-based COVID-19 vaccines fall into a special class of therapeutic agents defined by the FDA as “gene therapy products” [36], such that recipient cells produce antigens for transmembrane expression, or to leave the cell, to secondarily invoke an immune response. By design, therefore, by employing virus-like invasion and hijack of cellular transcription, both mRNA and adenovectorDNA gene-based vaccines cause non-immune cells to become de facto antigen-presenting cells, in their mode of immunogenicity. Therefore, these novel vaccine platforms risk tissue damage secondary to cytopathic autoimmune responses, raised against cells expressing foreign spike antigens.

Before the SARS-CoV-2 pandemic, the use of such technology was experimental and mostly restricted to making proteins for the therapy of metastatic cancer. No mRNA vaccines had ever been authorised for public usage prior to the COVID-19 pandemic [37] and viral-vectorDNA vaccines only had limited use for Ebola, Dengue, and Japanese encephalitis [38].

Documents obtained under a Freedom of Information (FOI) request reveal the mRNA COVID-19 vaccines were developed via the Trump Administration’s “Operation Warp Speed” program under the auspices of the US Department of Defense. The gene technology vaccines were emergency “countermeasures” to a national security threat, which arguably the pandemic at first appeared to be in 2020. As such, many of the FDA’s normal, protracted, and time-consuming safety testing and toxicology protocols were bypassed, in the rush to Emergency Use Authorisation status [39,40,41].

### 6.2. Wide Distribution of Lipid-Nanoparticle

Turni and Lefringhausen [42], in “COVID-19 vaccines—An Australian Review”*,* note that the lipid-nanoparticle, the carrier for synthetic mRNA, is potentially inflammatory in its own right, crosses membranes and distributes widely in the body. It crosses both the blood-brain barrier and the blood-placenta barrier. They cite the EMA report on the Moderna vaccine “that mRNA could be detected in the brain following intramuscular administration at about 2% of the level found in plasma” (p. 491). They also cite research [43,44,45] that describes how and why lipid-nanoparticles easily traverse the blood-brain barrier.

A/Prof Byram Bridle, Canadian virologist-vaccinologist, obtained Pfizer rodent study biodistribution data from the Japanese Pharmaceuticals and Medical Devices Agency (PMDA) via a FOI request in 2021 [46]. Judicial Watch, a US independent watchdog foundation, obtained the same Pfizer study report via FOI lawsuit to the US Department of Health and Human Services after the FDA and CDC refused to comply [47]. A more recent FOI request to the Australian TGA (FOI reply 2389-6), reveals on page 45 of the TGA’s “nonclinical evaluation report: BNT162b2 COVID-19 vaccine” that the same study was part of the TGA’s evaluation in January 2021 prior to its provisional authorisation [5] (p. 45). 

**Figure 5:**
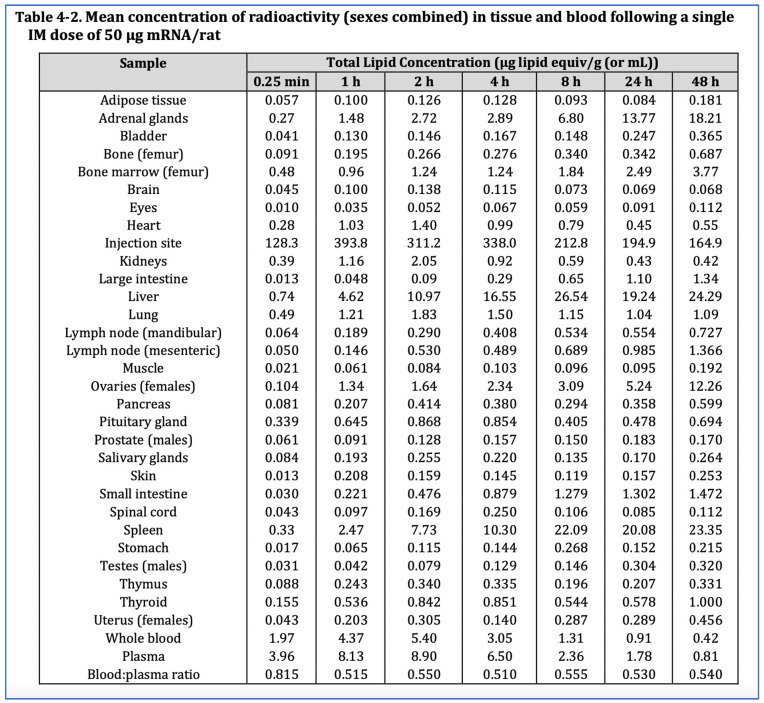
Biodistribution of lipid-nanoparticle in rat, Pfizer study November 2020. From TGA FOI reply 2389-6 [5] (p. 45).

The Pfizer biodistribution study involved 63 Wistar Han rats of whom 42 (21 male, 21 female) were injected with the human equivalent of 50 µg mRNA per animal, and an additional 21 male rats were injected with the equivalent of a Moderna COVID-19 vaccine dose of 100 µg mRNA per animal. The mRNA coding for Luciferase was encapsulated in liquid nanoparticles containing radiolabelled cholesterol, injected into the gluteal muscle and monitored for 48 h. As indicated in Figure 5, the biodistribution data showed the lipid-nanoparticles, which were designed to pass easily through biological tissues and membranes, travel to all organs. By 48 h, 75% had left the injection site for elsewhere [5,47].

Although the highest levels went to the spleen and liver, where high cell turnover helps timely repair of any cytotoxic damage, the lipid-nanoparticle, and by implication the mRNA, went to seemingly all organs, particularly the ovaries and adrenal glands but also the brain, eyes, heart, testes, uterus, pituitary gland, spinal cord, thymus, bone marrow.

The Pfizer rat biodistribution study has been corroborated. Chinese researchers injected mice with lipid-nanoparticle-mRNA complexes (mRNA-LNPs) encoding the firefly luciferase gene and biodistribution from the injection site “became rapidly distributed throughout the body with a large presence in the liver” and the “non-linear relationship between the LNP exposure and the protein expression level varies in different tissues and organs” [48] (p. 114). Smaller mRNA-LNP complexes transfected further and relatively smaller amounts of mRNA in the liver and lymph nodes produced higher rates of encoded bioluminescent protein than at the injection site muscle. The authors stated:

“*The duration and kinetics of transgene expression are affected by the pharmacokinetics and biodistribution of the delivery systems. The pharmacokinetic-pharmacodynamic relationship of mRNA-LNPs is highly complex, making the prediction of gene expression and efficacy (pharmacodynamics) unlikely just based on LNP exposures in tissue (pharmacokinetics)*”.

[46] (pp. 112–113)

Effectively the lipid-nanoparticle, and presumably its mRNA payload, distributes throughout the whole body and gene expression varies unpredictably [5,46,48]. 

### 6.3. Long-lasting Pseudouridine mRNA

Natural messenger RNA is highly unstable, so the synthetic mRNA that codes for spike protein in Moderna and Pfizer COVID-19 vaccines has been stabilised by replacement of uridine with N1-methylpseudouridine [37]. This intervention is now known to make the synthetic mRNA excessively stable over a prolonged period [49]. Fertig et al. [50] found the lipid-nanoparticle and contained mRNA were still circulating in blood plasma 15 days post-vaccination. Recent research found the mRNA in blood plasma at 28 days post-vaccination [51]. Also, the S1 subunit was found recirculating in picomolar amounts along with full spike protein in a Brigham and Women’s Hospital study of 13 nurses vaccinated with the Moderna COVID-19 mRNA vaccine to about 42–72 h [52].

Röltgen et al. [53] found persistence for the full 60 days duration of their study of both mRNA and free spike proteins in the cytoplasm and nuclei of germinal cells in axillary lymph nodes ipsilateral to deltoid muscle injection site. Spike protein persisted in 96% of vaccinees blood up to 2 days post-vaccination and was still present in 63% of vaccinees 1 week after the first dose. After the second dose, the detection of spike protein “is impeded … likely due to … anti-spike antibodies” (p. 1037). However, as shown earlier the modified RNA molecules are extraordinarily stable, and as long as they persist inside the cell, and the cell is not attacked and killed by the immune system, intracellular ribosomal spike protein production will persist. No studies have determined the stability of the vaccine-induced spike protein, but free spike protein has been found circulating up to 19 days post-vaccination in the plasma of young individuals with post-vaccine myocarditis [54].

The implications of Röltgen et al. [53] findings have been elaborated in detail in a blogpost by Jikomes [55] as indicative of danger, whereas a blogpost by Yong [56] argues the prolonged presence of mRNA and spike proteins is not dangerous. However, Yong concedes the persistence was unexpected. Health regulatory authorities had assured clinicians and the public early in the COVID-19 vaccine rollout that the persistence of mRNA spike protein production would be brief and localised to the deltoid. This is clearly not the case and the biological implications of persistent translation of spike protein within multiple tissue types warrant investigation.

The findings of these studies are consistent with the 14-day half-life for the mRNA-LNP in the Japanese Ministry of Health Pfizer rat biodistribution study [46] and are summarised in Table 1.

Cells that take up mRNA from the mRNA vaccines package some of the mRNA with ionizable cationic lipids into small lipid particles that are released as exosomes [59]. Other research has found spike proteins persist in circulating exosomes for at least four months after Pfizer COVID-19 vaccination [57]. This shows spike protein endurance, like mRNA endurance, is long-lasting in vivo. Varicella zoster virus (VZV) reactivation as shingles is the most common cutaneous adverse event after COVID-19 mRNA vaccination, and a case has been reported in which spike protein was detected in skin lesions 3 months after vaccination [58]. These authors postulated that:

“*mRNA COVID-19 vaccination might induce persistent VZV reactivation through perturbing the immune system, although it remained elusive whether the expressed spike protein played a pathogenic role*”.

[58] (abstract)

Several possible ways for COVID-19 vaccines to perturb the immune system are hypothesised by the authors—via the lipid-nanoparticles, N1-methylpseudouridine in mRNA, the spike protein (particularly the S1 subunit), antibody-dependent enhancement and overwhelming antigenic stimulus [58]. Our review of a large and growing literature reveals these concerns to have an evidentiary basis, and there to be a pathogenic role for the spike protein.

### 6.4. Nanoparticle Toxicology

Wang et al. showed in 2018 that even small amounts of nanoparticles taken up via lungs or skin can lead to cytotoxic effects [60]. When ingested, nanoparticles target predominantly the mesenteric lymph nodes, liver, and spleen, while when injected as a drug carrier, they can pass any barrier and translocate to the brain, ovaries, and testis, mainly after phagocytosis by macrophages which help distribute them across the body. Reproductive toxicity effects beyond the scope of this review.

The molecular mechanisms involved in nanoparticle toxicity to the reproductive system are not fully understood, but possible mechanisms include oxidative stress, apoptosis, inflammation, and genotoxicity through induction of reactive oxygen species (ROS), causing damage at the molecular and genetic levels which results in cytotoxicity and DNA damage. 

Of particular concern in mRNA-LNP complexes are the two propriety functional excipients, ALC-0315 and ALC-0159, never before used in a medicinal product and not registered in either the European Pharmacopoeia or in the European C&L inventory [61]. A question in the European Parliament in December 2021 noted that “Echelon, the manufacturer of these nanoparticles, specifies they are ‘for research only and not for human use’”. The reply on behalf of the European Commission was that the excipient “in Comirnaty has been demonstrated to be appropriate … in compliance with the relevant EMA scientific guidelines and standards” [62]. Despite this reassurance, the presence of electrolytes in the preparation and manual dilution before inoculation raises serious questions about the stability of the resulting suspension and the polydispersity index of the nanomaterials contained in it, factors that can be hypothesised as the root causes of numerous post-vaccination adverse effects. 

A nanoparticle in solution forms a colloidal system whose stability prevents the aggregation of particles through electrostatic repulsion. The parameter used to calculate colloidal stability is the Zeta potential, which refers to the potential generated by a double layer of electric charges. When the potential is low, attractive forces prevail over repulsive and more aggregates will form. The stability of a colloidal biphasic system is a precarious balance dependent upon ratios, processing methods, correct temperatures, and the presence of electrolytes [63]. After dilution with sodium chloride solution, the final ratio in Comirnaty is 2.61 mg of electrolytes versus only 0.48 mg of ALC-0315 + ALC-0159. This can only lead to a drastic reduction in the Zeta potential, with predictable aggregation, agglomeration, and, finally, flocculation. One can postulate the damage caused by aggregation of nanoparticles in capillaries throughout the body.

Should the colloidal suspension stay stable enough to disperse in lymph and blood, the nanoparticles as well as their toxic load will distribute across the body, cross blood-brain, blood-placental and other biological barriers and likely cause cell death and inflammation wherever they accumulate. Additionally, the elimination of toxic nanoparticles from the body is not straightforward. Particles of 5.5 nm or less can be excreted after glomerular filtration in the kidneys via the urinary tract. Larger particles could in theory be broken down going through the hepatobiliary tract, however, tend to be bound by, e.g., Kupffer cells, the resident macrophages, which slows down their processing considerably [64]. The mRNA-LNP complexes are around 100 nm in size and well above the size which allows their elimination via the kidneys. This would account for their accumulation in the liver and the observed liver toxicity.

### 6.5. Lipid-Nanoparticles Are Pro-Inflammatory

The lipid-nanoparticles used in the COVID-19 vaccines have been found to induce significant inflammatory cytokine secretion and macrophage inflammatory proteins with cell death [43]. Ndeupen et al. [43] note this pro-inflammatory effect of the lipid-nanoparticles would increase the vaccine adjuvant immunogenicity of the COVID-19 mRNA vaccines and add to the adverse events. The authors did not consider the widespread biodistribution of the lipid-nanoparticle, and therefore the potential for wide-ranging serious COVID-19 vaccine adverse effects across organs and systems.

Trougakis et al. [65] reviewing literature on adverse events from COVID-19 mRNA vaccines, noted the risk of spike protein-driven pathology, which they termed the “spike hypothesis”. However, Trougakis and colleagues also reviewed evidence of lipid-nanoparticles’ pro-inflammatory properties from animal model studies. These include “activating Toll-like receptors, excessive neutrophil infiltration, activation of diverse inflammatory pathways, and production of various inflammatory cytokines and chemokines” [65] (p. 544).

Hence, even if one were to change the antigen expressed there would likely still be adverse events. Halma et al. [66] point to the changes made to the mRNA and the ingredients of the lipid-nanoparticles, especially the addition of polyethylene glycol (PEG), that made it both more resistant to degradation and helped it to evade the immune system with lipid-nanoparticles helping biodistribution and bioaccumulation. Bioaccumulation can lead to blockage of small blood and lymphatic vessels. Biodistribution means that cell death and inflammation could occur in all organs including the brain, placenta, and testes, as has been seen with the COVID-19 mRNA vaccine [5,44,45,46]. 

PEG is known to cause anaphylactic reactions in some people, which is stated as a known adverse event in the vaccine patient information leaflet. Beside lipid-nanoparticle-encapsulated mRNA being highly inflammatory, antibodies against the spike protein damage cells and tissue that produce the spike protein. Regardless of which antigen is produced, damage to cells will occur in an autoimmune reaction [67]. 

Mechanisms involved in autoimmune damage to cells producing an endogenous protein include the development of cross-reactivity to the endogenous protein [68], immune-mediated toxicity [69], and immune tolerance due to switching to IgG4 [70]. Switching to an IgG4 immune response has consequences for cancer susceptibility [71], pregnancy [72] and IgG4-related diseases, which are chronic inflammatory conditions [73].

Another risk, and problematic with prior vaccines against coronaviruses both in the human and veterinary field, is the risk of antibody-dependent enhancement [66]. 

### 6.6. Novavax COVID-19 Vaccine Toxicity and Novel Lipid-Nanoparticle Technology

That lipid-nanoparticle biodistribution makes an important contribution to adverse events is further suggested by adverse event reports from the protein-based Novavax COVID-19 vaccine Nuvaxovid. It has the novel technology of a lipid-nanoparticle matrix which could potentially increase biodistribution of the unmodified spike protein, with intact furin cleavage and receptor binding domain sites. In response to a query about biodistribution studies, Novavax replied in mid-2021 that “a pharmacokinetic/pharmacodynamic study has not been performed on the Novavax COVID-19 vaccine” (personal communication Novavax-Parry, 30 July 2021). 

Myocarditis adverse events have been reported for the Novavax COVID-19 vaccine in several nations including New Zealand, where the regulator has released an “Alert Communication” on myocarditis [74]. This suggests a pathogenic amount of spike proteins from the Novavax COVID-19 vaccine can on occasion reach the heart. Overall, the adverse event reports from the Novavax COVID-19 vaccine are less than from the gene-based vaccines, which would be consistent with a dose-response effect for spike proteins. However, the lipid-nanoparticle matrix itself may be responsible for some of the myocarditis reports.

### 6.7. AstraZeneca COVID-19 Vaccine Biodistribution Data

In October 2022 a FOI request (MHRA IR07151D) obtained AstraZeneca documents that had been submitted to the British MHRA. According to the AstraZeneca “Nonclinical Overview” dated 21 December 2020, the rationale for initially not performing biodistribution studies on the AstraZeneca adenovirusDNA COVID-19 vaccine was that prior studies on viral vector vaccines showed minimal spread from the deltoid muscle and axillary lymph nodes to distal organs [75]:

“*The biodistribution of AZD1222 following intramuscular administration is expected to be similar to that of AdCh63, confined to the site of injection and draining lymph nodes*”.

[75] (p. 13)

However, a later AstraZeneca “Nonclinical Overview” dated 26 April 2021, which included new mouse biodistribution studies on the company’s COVID-19 vaccine did reveal biodistribution to distal organs [6]:

“*The highest levels of AZD1222 vector DNA (103 to 107 copies/µg DNA) were observed in the intramuscular administration sites and sciatic nerve (close proximity to the administration sites) on Day 2. Lower levels of AZD1222 vector DNA (<LLOQ to 10^4^ copies/μg DNA) were observed in the bone marrow, liver, spleen and lung on Day 2. The levels of AZD1222 and the number of tissues with detectable levels of AZD1222 vector DNA decreased from Day 2 to 29, indicating elimination*”.

[6] (p. 14)

The document stressed that the viral-vector itself was not replicating as an adenovirus, but that misses the point of protein production of a toxic foreign antigen in bodily organs. Although this suggests lesser quantities of the viral-vectorDNA COVID-19 vaccines are widely biodistributed than with the lipid-nanoparticle carried modified mRNA COVID-19 vaccines, the capacity of the adenovectorDNA vaccines to produce significant quantities of spike proteins remains. An autopsy series of three cases of vaccine-induced immune thrombotic thrombocytopenia (VITT) with cerebral thrombosis related to the AstraZeneca COVID-19 vaccine found spike proteins in thrombosis and cerebral vein walls [7]. The authors state in the abstract:

“*SARS-CoV-2 spike protein was detected within the thrombus and in the adjacent vessel wall. Data indicate that neutrophils and complement activation associated with antispike immunity triggered by the vaccine are probably involved in the disease process*.”

### 6.8. Traditional COVID-19 Vaccines Not Contributing High Adverse Event Reports

Traditional vaccine technology COVID-19 vaccines are mostly available in non-Western nations [35]. These include inactivated virus vaccine technologies such as Covaxin manufactured by Bharat Biotech [76] in India, and CoronaVac made by Sinovac [77] in China. 

There are also traditional recombinant protein-based COVID-19 vaccines such as Spikogen, jointly developed by Australian and Iranian-based companies [78,79,80]. In Spikogen the spike protein antigen has been modified with the removal of furin cleavage site and RBD to reduce cell adhesion and entry and thus to reduce potential toxicity. A Spikogen phase 3 clinical trial in Iran involving 16,876 participants met its primary efficacy endpoint with greater than 60% protection against infection during a particularly widespread wave in Iran of the delta variant of SARS-CoV-2 [81,82]. Spikogen is on the market in Iran and recognised for travel to some nations including New Zealand, having been used for 8 million doses with no serious systemic adverse event reports to Iranian pharmacovigilance to date.

Traditional COVID-19 vaccines have not produced the high rates of adverse event reports that characterise the gene-based COVID-19 vaccines. This is further evidence that the risk is in the body-wide biodistribution and prolonged production of spike proteins. It points to pathogenicity of the spike protein and, given the evidence described above, also the lipid-nanoparticle carrier matrix.

### 6.9. Autoimmune Risk of Foreign Antigens Presented by the Body’s Own Cells

As described above, evidence shows the spike protein to be innately toxic. Even if it were non-toxic in its own right, by virtue of its foreignness, spike protein could still produce pathophysiological damage through autoimmune responses. A straightforward consequence of a foreign protein. The lipid-nanoparticle matrix permits widespread biodistribution of mRNA gene codes to cells in most or all organs. The subsequent expression of the spike protein on cell surfaces, and as a soluble protein within the organs and blood stream, induces T-cell destruction of cells and tissues and B-cell antibodies. The latter may also cause immune complex deposition further damaging tissues via type III hypersensitivity.

Tissue damage, therefore, can be caused by the spike protein via autoimmune reactions, even if it is ‘non-toxic’. While this is of minor consequence in a muscle such as the deltoid, it causes serious and fatal adverse events when occurring in critical organs such as the brain, ovaries, and heart. The method of delivery—mRNA gene therapy via lipid-nanoparticles that traverse biological membranes—is a core problem and a key reason why this technology has never been commercially marketed, until now.

The fact Moderna and other big pharmaceutical companies plan large-scale mRNA vaccine manufacture for many other diseases, in the absence of a full and detailed inquiry, is, therefore, deeply troubling.

### 6.10. Pathophysiology of Virus and Vaccine Spike Protein

The natural course of new pandemic/epidemic viruses is to become more infectious and less pathogenic with time. This has demonstrably been the case with SARS-CoV-2 where the original Wuhan strain and subsequent alpha and other early variants were quite pathogenic, the delta variant spread more easily but was somewhat less pathogenic, and the various omicron subvariants have been highly infectious but even less pathogenic in illness severity. In particular, the omicron subvariants have targeted the upper respiratory tract rather than the lower respiratory tract, leading to less systemic penetration of the virus and the spike protein [83].

On the other hand, the mRNA and adenovectorDNA vaccines cause human cells to manufacture a slightly modified version of the original Wuhan strain spike protein. Some “bivalent” booster doses add genetic code for omicron variant spike protein [84,85]. If an individual suffers wide biodistribution of this genetic code, many more spike proteins can be produced systemically than generally occurs with the natural virus. This is more likely for anyone who is young and healthy. The elderly and those with comorbidities have a greater risk of serious SARS-CoV-2 viral infection deep in the lungs and systemically, whereas the young and healthy tend to rid themselves of the virus in the upper respiratory mucosa. Therefore, in the young and healthy the encoding-based COVID-19 vaccines will transfect a far more diverse set of tissues than infection by the virus itself.

Many studies have demonstrated the spike protein is toxic. In “Understanding the Pharmacology of COVID-19 mRNA Vaccines: Playing Dice with the Spike?”, Cosentino and Marino (2022) reviewed the evidence for the toxicity of the spike protein [86]. They argued that the COVID-19 mRNA vaccines should rightly be described as “prodrugs” as they meet the dictionary definition: “a pharmacologically inactive substance that is converted in the body (as by enzymatic action) into a pharmacologically active drug”. This occurs via the mRNA action in ribosomes to cause the synthesis of the spike protein [86] (p. 3).

Cosentino and Marino (2022) reviewed the evidence for widespread biodistribution of the mRNA and concluded that “evidence strongly supports the possible link between inappropriate expression of S protein in sensitive tissues and subsequent tissue damage” [86] (p. 2). 

They reviewed the literature on the pharmacology and pathophysiological effects of the spike protein on bodily tissues, which include [86] (p. 4–5):

- Binding to ACE-2 receptors as a “potential trigger for platelet aggregation, thrombosis and inflammation, as well as for hypertension and other cardiovascular disease”.
- Disruption of CD147 transmembrane glycoprotein which interferes with cardiac pericyte and erythrocyte function may result in myocarditis, haemolytic anaemia, blood hyperviscosity, and possibly neurodegenerative processes.
- Binding to Toll-like receptors 2 and 4 (TLR2, TLR4), with theoretical pathogenic effects via increased inflammatory cytokine cascades, due to (1) activation of Nuclear Factor kappa B (NF-κB pathway) and deficient macrophage immune function via TLR2, and (2) lung damage, myocarditis and multiorgan injury via TLR4, that had yet to be properly investigated by the world’s research community.
- Binding to the high affinity oestrogen receptor alpha (ER alpha) is possibly responsible for the menstrual irregularities commonly observed after COVID-19 vaccination and raising concerns of potential involvement in breast cancer.
- Spike protein S2 subunit specifically interacts with proteins p53 BP1 and BRCA1. The p53 BP1 is a well-established tumour suppressor; the BRCA1 is frequently mutated both in breast cancer and in prostate cancer [87].

Cosentino and Marino noted that these “potential toxicological issues” were not “taken into consideration in the studies that led to the marketing authorisation, precisely because … these products were treated as conventional vaccines”, when in fact they are gene insertions acting as prodrugs [86] (p. 5). 

In vitro research found the receptor binding domain (RBD) of the spike protein (the S1 unit) was the most active agent to trigger a pro-inflammatory response from dendritic cells [88].

Further in vitro research with human pulmonary artery muscle and endothelial cells treated with full-length spike protein or the RBD alone, found in this case the RBD to be relatively inert, but the full-length spike protein to induce enlargement of the pulmonary vessel cells via phosphorylation of protein MEK (mitogen-activated protein kinase kinase) [89]. This was found to also be the case in vivo when intratracheal administration of the S1 unit/RBD into transgenic mice with human ACE-2 on their cells showed a dramatic increase in inflammatory cytokines in bronchial lavage fluid from mice who received the spike protein S1 unit, whereas this was minimal for control mice (intratracheal saline) and mild and late for whole spike protein administered mice, indicating the cleaving of the S1 (RBD) unit increases the ACE-2 associated pathology [90]. 

Injection of mice, bred to have human-like ACE-2 receptors with spike protein S1/RBD unit was found to induce COVID-19-like acute pulmonary pathology, indicating it is the spike protein, unless modified as in the Australin-Iranian vaccine Spikogen [78,79], that is a cytotoxin primarily responsible for the severity of the SARS-CoV-2 respiratory infection [86]. This, in retrospect, means it has been a particularly poor choice for vaccine development purposes.

In a preprint, McKernan et al. [91] quantify the pharmacokinetics of the mRNA vaccines as creating greater numbers of spike proteins than the SARS-CoV-2 virus, and more systemically in most people not prone to overwhelming COVID-19 viral infection:

“*The pharmacokinetics of injection are different from an infection; 30–100 µg per injection (90–300 µg for those boosted) of Spike mRNA equates to 13 trillion to 40 trillion mRNA molecules injected in a few seconds with each injection. The pharmacokinetics of this bolus injection differs from that of viral replication that occurs over the course of a few days. If each of these mRNAs can produce 10–100 spike proteins and you have 30–40 trillion cells, there may be a far greater systemic quantity and a much longer duration of spike protein exposure through the vaccination route than natural infection*”.

[91] (p.12)

Human tissue production of antigens means that the dose is likely to vary between individuals. This will be for reasons of individual genetics and physiology, the tissues exposed to the code, batch and vial variability of the product and manner of transportation, refrigeration, and administration. In terms of the toxicological principle *dosis sola facit venenum* (the dose makes the poison), this aspect on its own casts doubt on the safety of mRNA and viral vector DNA vaccines.

Around the time the COVID-19 vaccines were released to the public, researchers at the Salk Institute found that the SARS-CoV-2 virus relies upon the spike protein binding to ACE-2 receptors on host cells to gain cell entry [92]. ACE-2 is protective in the cardiovascular system, and SARS-CoV-2 spike protein promotes lung injury through a decrease in the level of ACE-2. The Salk Institute team showed that the spike protein alone can damage vascular endothelial cells by downregulation of ACE-2, inhibition of endothelial nitric oxide synthase (eNOS), impairment of mitochondrial function and direct impairment of endothelial function.

### 6.11. Disruption of the Nicotinic Cholinergic Anti-Inflammatory Pathway

High doses of the toxin-like spike protein binding domain (RBD) inhibit acetylcholine (ACh)-induced α7 nAChR responses. Inhibition of these α7 nACHRs has profound effects [33]. The nicotinic cholinergic system has been labelled the ‘Cholinergic Anti-inflammatory Pathway’ (CAP), as the activation of these receptors controls inflammation and their inhibition results in uncontrolled inflammation. The CAP forms a multi-faceted network, with distribution in neuronal and non-neuronal cells, and diverse functions throughout the body. In addition to the nervous system, α7 nAChRs are expressed in non-neuronal cells such as lymphocytes, monocytes, macrophages, dendritic cells, adipocytes, keratinocytes, endothelial cells, and epithelial cells of the intestine and lung. With such widespread distribution, nAChRs could be implicated in the pathophysiology of severe COVID-19 via mechanisms, both through and independent of the cholinergic anti-inflammatory pathway [32].

The modulation of inflammatory and immune response by the CNS through the vagus nerve is based on bi-directional communication between the immune and nervous systems. Afferent vagus nerve fibres, located in nucleus tractus solitarius, provide sensory input to the CNS about the inflammatory status that can result in the transmission of efferent signals, originating from the dorsal motor nucleus, to control the inflammatory response. Such a response is rapid and localised, unlike the diffusible anti-inflammatory network, which is slow, distributed, non-integrated and dependent on concentration gradients [32].

Activated via the vagal nerve release of ACh, nACHRs are found in the immune system on T-cells, B-cells, macrophages, monocytes, neutrophils and mast cells and act to reduce inflammation, including the reduction of proinflammatory cytokines, such as IL-6, while promoting anti-inflammatory cytokines such as IL-4 [93]. Dysregulation of nAChR by SARS-CoV-2 could also suppress the counterbalance to the sympathetic nervous system and thus promote the central sympathetic drive and the development of the sympathetic-driven cytokine storm [94]. In turn, the sympathetic storm triggers oxidative stress and hyperinflammation by increasing the generation of reactive oxygen species (ROS) and the release of pro-inflammatory cytokines.

NAChR are also found in the respiratory tract. Subtype α3β4 nAChR support cilia function and mucociliary clearance, and α7 nAChR stimulation is anti-inflammatory. Hence, the inhibition of both these receptor types, as spike protein is able to do, would contribute significantly to the lung pathology seen in both acute COVID-19 and long COVID [95]. 

SARS-CoV-2 infection-induced stress and suppression of the cholinergic pathways via nAChR inhibition may also activate the sympathetic nervous system (SNS) leading to neuro-hormonal stimulation and activation of pro-inflammatory cytokines with further development of a sympathetic storm. Sympathetic over-activation in COVID-19 is correlated with an increase in capillary pulmonary leakage, alveolar damage, and the development of acute respiratory distress syndrome. Furthermore, SARS-CoV-2 can spread through pulmonary mechanoreceptors and chemoreceptors to the medullary respiratory centre in a retrograde manner resulting in sudden respiratory failure as a result of nAChR inhibition in the parasympathetic medullary centres [96]. 

Once someone is infected with SARS-CoV-2, the immune system is mobilised. As the virus replicates, cell and viral debris or virions may interact with the nAChRs to block the cholinergic anti-inflammatory pathway. If the initial immune response is not enough to combat the viral invasion at an early stage, the extensive and prolonged replication of the virus will eventually disrupt the cholinergic anti-inflammatory pathway and seriously compromise the ability to control and regulate the immune response. The uncontrolled action of pro-inflammatory cytokines will result in the development of cytokine storm, with acute lung injury and acute respiratory distress syndrome (ARDS), coagulation disturbances and multiorgan failure. Based on this hypothesis, COVID-19 appears to eventually become a disease of the nicotinic cholinergic system [92].

This same mechanism can explain both the breadth and severity of symptoms experienced in long COVID and in COVID-19 vaccine injuries. The former shows failure to clear spike protein and virus, with uncontrolled immune activation and sequelae [97], and in the latter vaccine injuries, where spike protein overwhelms the system and is produced for months, there is increased load with each subsequent injection. This also provides a mechanism for possible interventions with α7 nAChR agonists and positive allosteric modulators (PAMS).

## 7. Evidence of ‘Spikeopathy’—Spike Protein Pathogenicity

The spike protein of SARS-CoV-2 has turned out to be pathogenic. The term “spikopathy” has been coined [98] as its pathological effects, like tuberculosis, appear to be legion, widespread in body organs, and induce a myriad of known diseases and syndromes. The term is spelled “spikeopathy” by others on the internet and we have chosen that spelling.

**Figure 6:**
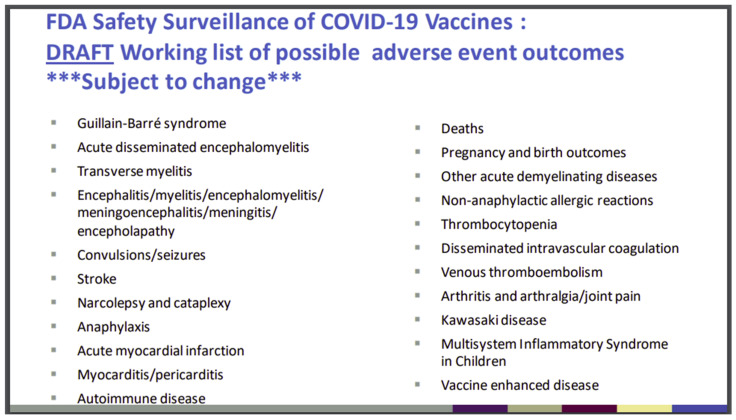
Slide 16 FDA’s VRBPAC meeting, Oct. 2022 [99].

Figure 6 shows the FDA was aware of this potential before the public release of the gene-based COVID-19 vaccines. It is the 16th slide from a PowerPoint presentation of the “Vaccines and Related Biological Products Advisory Committee (VRBPAC) 22 October 2020, Meeting” [99]. What is striking is the predictive accuracy of these mostly neurological, cardiovascular, and autoimmune “possible adverse events” with those reported to VAERS and other global vaccine injury databases.

The website www.react19.org lists as of June 2023 over 3400 published papers and case reports of COVID-19 vaccine harms under over twenty organ system and syndrome headings [100]. Here, we will review some key organ systems in relation to the pathogenic effects of the COVID-19 mRNA and adenovectorDNA-produced spike proteins.

### 7.1. Cardiovascular Pathogenesis

Literature accumulates about the cardiovascular harms of COVID-19 vaccines. For example, as of June 2023 react19.org, under the heading “Cardiac”, lists 432 peer-reviewed papers and case reports covering myocarditis, cardiomyopathy, myocardial infarction, hypertension, aortic dissection, postural orthostatic tachycardia syndrome (POTS), tachycardia, and conduction disturbance [100].

#### 7.1.1. Myocarditis and Pericarditis

Reports of myocarditis and pericarditis are particularly numerous. Yonker et al. [54] found free spike proteins in the blood of 16 adolescents and young adults who developed post-vaccination myocarditis, but not in 45 post-vaccination age-matched controls without myocarditis. The authors examined immuno-profiles and free spike protein plasma concentrations in young subjects with myocarditis after vaccination with COVID-19 mRNA vaccines. Significantly elevated full-length free spike protein concentrations, unbound to antibodies, were found in the myocarditis patients compared with controls. Antibody profiles and T-cell responses were similar between subjects with myocarditis and carefully age-matched controls, but it may be reasoned that part of the variance seen with regard to myocarditis as a complication of mRNA vaccination, may be explained by the fact that some achieve greater transcription and secretion into the blood. This raises serious concern about the pathogenicity of free spike protein in such cases of myocarditis.

Avolio et al. [101] found the free SARS-CoV-2 spike protein, separated from the virus, could cause microvascular disease via several mechanisms, which include stimulation of cardiac pericytes to engage in pro-inflammatory cytokine production via CD147 receptor binding. Further evidence for the pathogenicity of spike protein is from mouse studies where spike protein-induced cardiac fibrosis and myocardial contractile impairment may underlie COVID-19-related cardiomyopathy [102].

The possibility that COVID-19 vaccine-associated myocarditis, as opposed to the hypersensitivity myocarditis seen with agents such as the smallpox vaccine, is in fact autoimmune, is considered by Baumeier and colleagues [103] in a series describing 15 cases with endomyocardial biopsies (EMB), a study is discussed in a later section of this paper. Like other studies and case reports, lymphocytic infiltration was seen in association with intracardiac spike expression (although the authors did not specifically refer to lipid-nanoparticle biodistribution characteristics). 

Barmada et al. [104], in a recent study from Yale in light of the findings of Yonker et al. [54] and Baumeier et al. [103] consider whether spike-induced molecular mimicry is the driver of autoimmune myocardial attack. They effectively exclude this possibility in a serum study by employing REAP, a “rapid extracellular antigen profiling screen” for autoantibodies. They additionally postulate “cytokinopathy”, with reference to serum cytokine profiles and other markers of inflammation in a subgroup, but do not report blood concentrations of spike protein, or obtain myocardial tissue. 

From the above, although much laboratory study remains to be conducted regarding the myocardial inflammation seen prominently after mRNA vaccinations, it appears that spike protein plays a role. Whilst molecular mimicry is not the reason, direct toxic effects of spike protein may be implicated, in addition to the reaction of the immune system to the presence of spike protein, either expressed in or deposited in the myocardium. That myocarditis is precipitated by spikeopathy is further indicated in that the adenovectorDNA COVID-19 vaccines of both AstraZeneca and Johnson & Johnson, as well as the Novavax protein-based lipid-nanoparticle embedded vaccine, have been reported as causative [105,106].

How common is COVID-19 vaccine-induced myocarditis and pericarditis? As a baseline, a study published on 7 January 2020, the eve of the SARS-CoV-2 pandemic, reported: “viral myocarditis has an incidence rate of 10 to 22 per 100,000 individuals [107]. 

As to community epidemiological incidence, a review in the *New England Journal of Medicine* [108] noted that the annual incidence rate depended on the level of investigation:

“*Before the COVID-19 pandemic, the estimated global incidence of myocarditis was 1 to 10 cases per 100,000 persons per year (12). The highest risk was among people between 20 and 40 years of age and among men; 6.1 cases per 100,000 men and 4.4 cases per 100,000 women. The increased use of cardiac MRI has led to a gradual rise in the reported incidence of myocarditis in the United States, from 9.5 to 14.4 cases per 100,000*”.

[108] (p. 1488)

Health authorities like the FDA, TGA and other regulators have claimed that post-COVID-19 vaccination myocarditis is very rare. An early study of 2.39 million Kaiser Permanente insured Californian adults who received at least one dose of a Pfizer or Moderna COVID-19 vaccine found only 15 cases of post-vaccine myocarditis, all males with a mean age of 25 years [109]. However, cases were based on physician reports to the Kaiser Permanente immunisation committee or hospitalised cases within 10 days of vaccination. Milder cases could have been missed; physicians might not always have reported cases to the committee.

A systematic review of pharmacovigilance reports to US VAERS, UK Yellow Card, and EU EudraVigilance databases up to March 16, 2022, found 18,204 submitted events of myocarditis and/or pericarditis, some fatal [110]. Given hundreds of millions of vaccine recipients, the authors noted this to be a rare event.

The FDA recognised the risk for myocarditis and pericarditis from the COVID-19 mRNA vaccines was real, especially in younger males after the second dose, but judged it to still be rare, and cited a VAERS-derived figure of 6.5 per 100,000 and up to 20 per 100,000 for adolescent boys [111]. The FDA did not calculate that pharmacovigilance databases, like its own FAERS (FDA Adverse Event Report System) and the CDC’s VAERS, have a large under-reporting factor. 

A common factor in this pharmacovigilance-derived estimate of the FDA, as well as those of others, is the failure to mention the perennial problem of underreporting in passive notification systems. Pharmacovigilance databases, like its own FAERS and the CDC’s VAERS, are acknowledged to have large under-reporting factors. As to how large the under-reporting factor is, is a matter of debate. 

Compounding the phenomenon of underreporting in the case of myocarditis, is that this diagnosis is difficult to make, and often depends on the availability of specialty units, cardiac MRI facilities and/or endomyocardial biopsy (EMB). The diagnosis can mimic myocardial infarction and thus can be misdiagnosed. In this regard, the paper by Baumeier et al. [103] (discussed later in this paper), noted that a third of those with histologically confirmed myocarditis, categorised as vaccine-associated on the basis of history and exclusion of other causative agents, did not have cardiac MRI evidence of myocarditis. Further, many cases of myocarditis are subclinical and may be missed in the acute phase. This does not mean, however, that a benign course is always expected since even minor fibrosis and scarring of the myocardium can create arrhythmogenic foci and may present later with serious and fatal arrhythmias, or else may eventually lead to heart failure (so-called inflammatory cardiomyopathy) [112]. Hence, it is rational to say that the exact frequency of vaccine-associated myocarditis is unknown: cases can be subclinical, missed, or misclassified and even specialised imaging may underdiagnose. 

An indication of how common subclinical myocarditis, or at least myocardial involvement, might be comes from a prospective study in Thailand. Adolescents (*n* = 301) with no cardiac history had cardiac biomarkers (troponin-T, creatinine kinase-band (CK-MB)), ECG, echocardiography, and diary of cardiac symptoms at baseline and on days 3, 7 and 14 after the second dose of Pfizer mRNA COVID-19 vaccine [113]. Although there was no control group, the diary, physical examination, and ECG results are of concern: “tachycardia (7.64%), shortness of breath (6.64%), palpitation (4.32%), and hypertension (3.99%)” (p. 4). Fifty-four adolescents (18%) had abnormal ECGs. Troponin elevation occurred in five adolescents, echocardiography detected pericardial effusions in three adolescents, and signs of myopericarditis in one adolescent led to ICU admission. In total seven adolescents presented “with myopericarditis, subclinical myocarditis, and pericarditis after second dose vaccination” but apart from the adolescent hospitalised to intensive care, the other six cases were subclinical or mild and easily missed if it were not for this rigorous prospective study [113] (p. 8, Table 3).

While this methodologically excellent Thai study appears not to have been replicated in terms of a full manuscript, a conference abstract suggested comparable results, with a simpler methodology [114]. Of 777 healthcare workers from University Hospital Basel who received a COVID-19 booster vaccination in late 2021 to early 2022, evidence of cardiomyonecrosis (troponinemia) was detected in 22 (2.8%), with no cause other than a Moderna COVID-19 mRNA-1273 booster injection [114]. Although in a different population, receiving the second mRNA vaccine dose, the Thai study reported a rate of 2.3% for myocarditis or pericarditis. Given that billions of doses have been given to the human population, this would equate to 2300 cases per 100,000. As all cases were male the rate was 3.5% for male adolescents [113].

Although the public health authorities’ narrative is that myocarditis from COVID-19 vaccines is mild and self-limiting, the evidence is that symptomatically while this might be the case, pathological changes in these young hearts are persistent. An Italian study followed 13 cases of post mRNA vaccine-induced myopericarditis, myocarditis or pericarditis, median age 15 years, for 12 weeks. Although overt symptoms in all but one case resolved, 12 of the 13 adolescents still had pericardial effusion and six of nine who had cardiac MRI scans had signs of “persistent, although decreased, myocardial injury” at the study end [115].

Subclinical myocarditis inducing cardiac fibrosis as foci for later arrhythmia under stress is a possible explanation for the epidemic of sudden deaths in youths and young to middle-aged adults since the advent of the COVID-19 vaccines [116,117]. This possibility was noted by the TGA early in the vaccine rollout [118]:

“*Even apparently mild episodes of myocarditis may lead to long-term sequelae, such as arrhythmias. … the majority of cases of myocarditis and/or pericarditis after mRNA COVID-19 vaccines (both Pfizer and Moderna) analysed to date occurred in older adolescents and young adults (aged 16 to 30 years), with the highest risk in younger males within days after dose 2*”.

[118] (p. 8)

#### 7.1.2. Thrombotic Effects of Spike Proteins

Somewhat separate to the pathogenesis of myocarditis, COVID-19 vaccine-induced spike protein binding to ACE-2 receptors can trigger platelet aggregation, thrombosis, and inflammation, thereby leading to blood clots [119,120]. Angeli et al. [99] summarise the biochemical pathway to these pathophysiological effects:

“*Free-floating Spike proteins released by the destroyed cells previously targeted by vaccines may interact with ACE-2 of other cells, thereby promoting ACE-2 internalisation and degradation (16,79). This mechanism may enhance the imbalance between Ang-II overactivity and Ang-1-7 deficiency through the loss of ACE-2 receptor activity, which may contribute to triggering inflammation, thrombosis, an increase in BP, and other adverse reactions (“Spike effect” of COVID-19 vaccines) (80,81). Moreover, the detrimental effects of other angiotensinases (POP and PRCP) deficiency on BP, thrombosis and inflammation are well supported*”.

[120] (p. 24)

The authors describe the mechanisms by which these clotting effects are more common in younger patients. Angiotensinases prolyl oligopeptidase (POP) and prolyl carboxypeptidases (PRCP) become deficient in older people with cardiovascular diseases and paradoxically this translates to less susceptibility to spike protein-induced cardiovascular pathogenesis, whereas with younger people the risk is increased:

“*The relative deficiency of POP and PRCP among young and healthy subjects does not counterbalance ACE-2 internalisation, downregulation and malfunction due to free-floating Spike protein interactions, resulting in an increased risk of Ang-II accumulation, and adverse reactions (“Spike effect” of COVID-19 vaccines)*”.

[120] (p. 26)

They also propose that pre-existing immunity from SARS-CoV-2 infection or prior vaccination induces greater immune responses to spike protein production by cells, such as platelets, endothelial vascular cells or myocytes, leading to increased inflammation and thrombogenic activity. In Angeli et al. [119] they conclude:

Whereas Phase III vaccine trials generally excluded participants with previous immunisation, vaccination of huge populations in real life will inevitably include individuals with preexisting immunity. This might lead to excessively enhanced inflammatory and thrombotic reactions in occasional subjects. Further research is urgently needed in this area.

Live electron microscopy has shown free spike proteins trigger platelets to deform and coagulate via filopodia induction and the interaction of spike protein with platelet integrins to cause coagulopathy [121]. Early in the pandemic, mice transfused with transgenic human ACE-2 receptor platelets developed thrombi due to spike protein binding with the ACE-2 receptors on the platelets [1]. The authors noted:

“*SARS-CoV-2 and its Spike protein directly stimulated platelets to facilitate the release of coagulation factors, the secretion of inflammatory factors, and the formation of leukocyte-platelet aggregates*”.

[1] (abstract)

The spike protein also was found to “competitively inhibit the bindings of antithrombin and heparin cofactor II to heparin/HS, causing abnormal increase in thrombin activity” [122]. In another mouse study, the spike protein was also found to “bind to the blood coagulation factor fibrinogen and induces structurally abnormal blood clots with heightened proinflammatory activity” and that “spike delays fibrinolysis” [123] (preprint).

French researchers at the Méditerranée Infection Institute in Marseille examined the effects of the spike proteins of the SARS-CoV-2 Wuhan, alpha, delta and omicron BA.1 variant on red blood cells (erythrocytes) in vitro and found the spike protein induced haemagglutination (clumping) of erythrocytes, the omicron BA.1 variant achieving this at lower concentrations down to 0.13 ng/µL, the earlier variants down to a concentration of 0.13 ng/µL. The mechanism of action was deduced via molecular modelling to be the positive charge on the spike protein reducing the natural electrostatic repulsion of negatively charged erythrocytes. Interestingly ivermectin when added to the in vitro solution bound strongly to the spike protein and prevented or reversed the haemagglutination depending on whether added pre or post the spike protein. The authors note the implications for treatment of vaccine adverse effects [124]. 

The plasma and internal membranes of other eukaryotic cells can be considered to function with anions and cations acting as a current loop to drive membrane potential on either side of the cell membrane [125,126]. The red blood cell’s unique design is a toroid where currents also flow on the surface of the torus. If this static flow on the erythrocyte membrane surface is interrupted by a lack of separation between the negative surface membrane charge and the Stern layer, the zeta potential weakening leads to distortion of shape, decreased electric permittivity, increased viscosity, flocculation and rheological alterations [127]. When this surface current flow is static, with efficient separation of the positively charged Stern layer and negative surface membrane charges, the zeta potential is enhanced and the size, shape, proportion, and curvature of the erythrocyte transforms to the optimal shape. Erythrocytes must maintain a biconcave discoid shape in order to efficiently deliver oxygen (O_2_) molecules and to uptake carbon dioxide (CO _2_) molecules [128]. The interpolation of a positive spike protein into the negatively charged erythrocyte membrane can thus be expected to result in significant alterations in erythrocyte shape and function.

Thrombotic complications of COVID-19 vaccination involve many potential mechanisms, such as endothelial cell damage, immune response, dysregulation of the renin-angiotensin-aldosterone system, and thrombo-inflammation. Additionally, platelets contain acetylcholine and express α7nAchR. Acetylcholine acts as an endogenous inhibitor of platelet aggregation. Hematopoietic α7nAchR deficiency increases platelet activation and, in experimental studies, α7nAchR stimulation can diminish the pro-inflammatory state and modulate platelet reactiveness via increased levels of nitric oxide (NO). Inhibition of platelet nAChR by SARS-CoV-2 thus promotes platelet hyperreactivity and thrombosis which are hallmarks of COVID-19 and vaccine injury [129]. 

These mechanisms would explain clotting to both the virus and the spike proteins produced by the gene-based COVID-19 vaccines. It also suggests that public health policies that negated natural immunity and mandated COVID-19 vaccination, and booster programs, put the young and non-elderly population at greater risk. To illustrate these increased risks of harms, blindness cases have been reported to pharmacovigilance databases and a recent large study of retinal vascular occlusion diagnoses in the United States from January 1, 2020, to December 31, 2022, found COVID-19 vaccinated individuals with Moderna, Pfizer or AstraZeneca COVID-19 vaccines had a 2.19 hazard ratio increased risk compared with unvaccinated Americans [130].

#### 7.1.3. Vaccine-Induced Immune Thrombotic Thrombocytopenia (VITT)

In contrast to the spike protein’s role in myopericarditis and thrombogenesis as described above, the syndrome of vaccine-induced immune thrombotic thrombocytopenia (VITT) seen with the adenovectorDNA vaccines of AstraZeneca and Johnson & Johnson, as well as the adenovectorDNA Russian Sputnik V vaccine [131], is a rare condition mediated by anti-PF4 platelet antibodies. It appears unrelated to the spike protein, and other components of the adenovectorDNA technology are being investigated [132]. It is odd that these vaccines have been mostly withdrawn from markets, whereas the mRNA COVID-19 vaccines with similar issues, albeit by different pathophysiological mechanisms, have not.

In addition to the presence of anti-PF4 antibodies in the pathogenesis of VITT, numerous authors have discussed the evidence for the role of NETosis (neutrophil extracellular traps) as a basis for thrombilia observed in adenoviral vector vaccines, independent of spike proteinaemia [133,134]. Interestingly, Talotta and Robertson discuss the possibility that NETosis may also play a part in the thrombophilic consequence of mRNA vaccines, noting for example that naked RNA, if escaping the confines of the LNP vector and leaked into the bloodstream, can act as a trigger for NETosis [135]. 

### 7.2. Autoimmune Disease

In 2020, prior to the launch of the vaccines, Lyons-Weiler suggested that more than one-third of COVID-19 proteins, the spike protein included, show problematic homology with key proteins in the human immune system. Thus, there is potential for autoimmune reactions against these proteins [8]. Vojdani et al. [9] cited Lyons-Weiler and went further in their testing, performing epitope mapping and applied monoclonal anti-SARS-CoV-2 spike protein and nucleoprotein antibodies to 55 human tissue antigens in vitro. They discovered that SARS-CoV-2 antibodies reacted with 28 of the tissue antigens, thus likely playing “a role in the multi-system disease process of COVID-19” (abstract) and may precipitate or exacerbate autoimmune diseases. Their paper was submitted in October 2020 and they noted historical precedents for vaccines causing autoimmune-related disorders and expressed concern that “an insufficiently vetted vaccine might mean trading freedom from COVID-19 to an autoimmune assault in the future” (p. 2).

Vojdani and colleagues found the 28 antigens had molecular mimicry/shared homology and reactivity with:

“*Gut and barrier proteins, gastrointestinal system cells, thyroid, nervous system, heart, joint, skin, muscle, mitochondria, and liver diseases*”.

[9] (p. 5)

Khavinson et al. in a paper titled “Homology between SARS-CoV-2 and human proteins” found more than two dozen heptamers and octamers, homologous with human proteins, some of which fuse to extended lengths along the length of the SARS-CoV-2 spike protein [136]. They noted that given the “structural similarity, a part of the immune response will be directed against the proteins of the host organism” (p.1).

Kelleni reports on the potential risk of vaccines to induce autoimmune diseases such as thrombocytopenia, myocarditis and immune-induced thrombosis and thromboembolism, all potentially fatal and possible causes of sudden death [137].

Most recently, a group from Saudi Arabia has found clear clinical emergence of autoimmune disease after COVID-19 vaccination. A series of exclusively new-onset autoimmune diseases are described after COVID-19 vaccination. The average time between vaccination and new onset disease was 7 days. Cases included vasculitis, neurological disease, systemic lupus erythematosus, inflammatory arthritis, and a case of Sjogren’s syndrome [138]. A systematic review by Rodríguez et al. documented 928 cases from 464 published reports of new or relapses of autoimmune disease following COVID-19 vaccination [139]. The authors noted:

“*The most common disease associated with new-onset events following vaccination were immune thrombocytopenia, myocarditis, and Guillain-Barré syndrome. In contrast, immune thrombocytopenia, psoriasis, IgA nephropathy, and systemic lupus erythematosus were the most common illnesses associated with relapsing episodes*”.

[139] (abstract)

Since Rodríguez et al.’s review, further peer-reviewed case reports keep appearing. A small sample includes autoimmune dermatological and vascular disorders attributed to the COVID-19 vaccines including IgA pemphigus where the “likely cause” was the AstraZeneca COVID-19 vaccine [140]; autoimmune bullous disease with IgG and IgM autoantibodies against epidermal basement membrane zone after mRNA COVID-19 vaccine [141]; polyarteritis nodosa in a 32-year-old male with “limb pain, fever, pulmonary embolism, and multiple subcutaneous nodules and haematomas” following the COVID-19 vaccine [142].

Autoimmune-related thyroid and renal case reports include that of Graves’ disease following the second dose of the Pfizer COVID-19 vaccine [143] and “rapidly progressive IgA nephropathy” following the third dose of the Moderna COVID-19 vaccine [144]. A 78-year-old woman developed IgG4-related sialadenitis and autoimmune pancreatitis following her second Pfizer COVID-19 vaccine, the authors concluded: “the use of mRNA vaccines requires more studies regarding their effects on the human immune system” [145] (p. 1550).

A 23-year-old woman suffered an ocular autoimmune reaction in the form of granulomatous anterior uveitis following her third Pfizer COVID-19 vaccine 15 days earlier [146]. The authors commented:

“*Autoimmune reaction in the uveal tissue via molecular mimicry as a result of an adaptive humoral and poly-specific cellular immune response against epitopes may be the potential mechanism for post-vaccine uveitis in this patient*”.

(p. 1034)

A case report of hepatitis-associated aplastic anaemia (HAAA) following COVID-19 vaccination in a 15-year-old girl from Japan [147] cited Talotta [148] and postulated:

“*The pathogenic mechanisms by which mRNA vaccination triggers the development of autoimmune disease remains unclear. mRNA vaccination triggers potential cross-reactivity between antibodies against the spike protein and self-antigens and may also activate immune responses, leading to the production of interferon I and other pro-inflammatory cytokines and chemokines*”.

[148] (p. 3)

Repeated antigenic stimulation of immunity, as occurs with the gene-based COVID-19 vaccines’ prolonged production of spike proteins, repeated booster doses and recurrent SARS-CoV-2 infections, has seen a rise in IgG4 levels over 480-fold above normal levels [149,150]. This IgG class shift can be associated with serious disease pathology relating to sudden cardiac death [151,152]. 

It has also been associated with Mikulicz syndrome with systemic involvement, pancreato-hepatobiliary disease, head/neck disease, retroperitoneal fibrosis/aortitis [153,154,155,156], as well as lymphadenopathy, sialadenitis, dacryoadenitis, autoimmune pancreatitis, periaortitis/retroperitoneal fibrosis, prostatitis, sclerosing cholangitis, sinusitis, inflammatory pseudotumour, mediastinal fibrosis, skin involvement, sclerosing thyroiditis, hypophysitis, orchitis and colitis [157,158,159,160]. 

These widespread autoimmune and pro-inflammatory effects of spike protein and potentially lipid-nanoparticles illustrate ‘spikeopathy’ to be another ‘great mimicker’ akin to tuberculosis, which makes the diagnosis of the underlying aetiology difficult [161].

### 7.3. Neurological Disorders

The most common group of adverse events reported from the gene-based COVID-19 vaccines to pharmacovigilance databases, including Pfizer’s own post-marketing research [162,163] is not cardiovascular but neurological. Neurological symptoms and cognitive deterioration with accelerated neurodegenerative disease are a feature of acute COVID-19, vaccination injuries and to some degree, long COVID [164]. 

As the lipid-nanoparticle carrier of the mRNA to make spike proteins crosses the blood-brain barrier, direct neurotoxic effects are possible [43]. Loss of blood-brain-barrier (BBB) impermeability has been demonstrated post-COVID-19 vaccination [165], and spike protein S1 can cross the BBB and translocate into the brain parenchyma [166,167]. Cell culture in vitro experiment of brain endothelial cells (a component of the BBB) showed the S1 subunit (RBD) bound to ACE-2 of endothelial cells to traverse the BBB. The S1 subunit correlated with mitochondrial impairment and also entered cell nuclei; the authors postulated that it could disrupt gene expression [168].

#### 7.3.1. Neurovascular and Neuroimmunological Aspects

To some degree, the pathophysiology is likely to be via vascular and autoimmune pathology in the central and peripheral nervous system. The spike protein has been found in in vitro human cell cultures to dysregulate brain vascular pericytes by increasing ACE-2 expression leading to these cells lining the cerebral vasculature and adopting a “contractile and myofibogenic phenotype” as well as a “potent inflammatory response” worsened by hypoxia [169].

In further mouse experiments infusion of spike protein into brains led to TLR4-mediated neuroinflammation and hippocampal microgliosis with associated memory dysfunction. It was noted in humans that cognitive dysfunction post-COVID-19 was more likely with a particular TLR4 genotype [170]. This replicated similar mouse experimentation finding that infusion of the S1 subunit (RBD) to mice hippocampus induced cell death and glial activation and the mice exhibited cognitive deficits and anxiety-like behaviour [171].

Tillman et al., (2023) [172] described how the co-expression of the S1 and S2 subunits of the SARS-CoV-2 spike protein, via a helical motif in the spike neck, causes profound downregulation of functional α7 nAChR, which “is implicated in neuropsychiatric diseases and disrupts the cholinergic anti-inflammatory pathway” (p. 689). German researchers [173] (preprint) autopsied mice that had been intravenously injected with the S1 unit of the spike protein and examined skulls from human autopsies. They found the S1 unit binding to cells in most organs, including ovaries and testes. In the brain they found the presence of S1 associated with differential expression of proteins, following the known expression of ACE-2 receptors, in the skull marrow, meninges and brain parenchyma compared to controls. S1 was seen in diverse regions of the brain, including the channels connecting skull marrow to the meninges (SMC) in both mice and humans. This suggests that in addition to the distribution of the S1 protein through phagocytic cells or direct extravasation from blood vessels, it could use these channels as pathways through the skull. The S1 protein accumulated in the marrow of tibia and femur as well as the spinal cord. 

Using human proteomics data, the authors found dysregulation of both complement and coagulation cascades concurring with known coagulopathies following injection. Neutrophil-related pathways were dysregulated, some proteins upregulated, and other proteins downregulated. Among the upregulated ones were proteins associated with inflammation, such as interferon-gamma (IFN-γ) and IFN-γ induced the protein C-X-C motif chemokine ligand 10 (CXCL10). Other protein changes were involved in neutrophil extracellular traps (NETosis) formation, neutrophil degranulation, and phosphatidylinositol 3-kinase/protein kinase B (PI3K-AKT) pathways. In the meninges, upregulated proteins were also associated with platelet activation, signalling, and aggregation. In the brain’s cerebral cortex, there were altered levels of ribosomal protein and dysregulation of neurodegeneration pathways. The plasma cytokines levels and plasma IL-6 were increased three days after injecting spike S1. 

In addition to experimentally injecting mice with the S1 unit of spike protein, they autopsied 34 patients who died from non-COVID-19 illnesses and found 10 of them had persisting spike proteins in their skulls and noted these might be involved in long COVID symptoms via their spread via the meninges into the brain parenchyma. In summary, the spike protein accumulates in various regions of the brain, persists there even after death, and causes activation of microglia, blocking of *α*7 nAChR and dysregulation of coagulation- and neutrophil-related pathways as well as upregulation of inflammatory proteins, all of which are connected to memory loss, inflammation of the brain and cell death [173].

Of note SARS-CoV-2 viral infection, especially the earlier variants, can cause loss of smell and thus shows neurotoxicity to the olfactory nerve. Mechanisms of neurotoxic action of the virus and the gene-based COVID-19 vaccines are subject to ongoing research.

Olajide et al. [174] proposed that the SARS-CoV-2 spike glycoprotein induces neuroinflammation via its effects on microglia by:

“*Induction of neuroinflammation by this protein in the microglia is mediated through activation of NF-κB and p38 MAPK, possibly as a result of TLR4 activation.*”

[174] (Abstract, p. 445)

“*Activation of BV-2 microglia by S1 resulted in the increased release of TNF-α, IL-6 and IL-1β, which are hallmarks of neuroinflammation. Activation of neuroinflammatory processes by the spike S1 protein was further confirmed by results showing increased iNOS-mediated production of NO by the protein in microglia. Elevated iNOS/NO has been previously linked to a wide range of CNS disorders including Alzheimer’s disease, Parkinson’s disease, multiple sclerosis, epilepsy and migraine*”.

[174] (p. 452–453)

In a cell culture in vitro experiment, spike protein has been implicated in increasing expression of alpha-synuclein (α-Syn), an aggregation-prone protein that is further implicated in the pathogenesis of Lewy bodies which are hallmark lesions in brains of patients with Parkinson’s Disease, Lewy body dementia and other neurodegenerative diseases [175].

Winkler et al. [176] caused mild respiratory COVID-19 in a mouse model expressing human ACE-2 in trachea and lung by exposure to SARS-CoV-2 intranasally. They detected no SARSCoV-2 in the brain but found signs of neuroinflammation as well as elevated levels of chemokines in cerebrospinal fluid and serum. These changes led to activation of microglia in subcortical and hippocampal white-matter regions. Microglia are commonly referred to as the macrophages of the central nervous system and maintain neuronal networks by removing dendritic spines and synapses during neuronal development. When activated in the mouse model, however, they transitioned to a neurotoxic state which in subcortical white matter led to loss of both oligodendrocyte precursors and mature oligodendrocytes. 

Additionally, myelin and myelinated axons decreased for at least 7 weeks after infection, impacting the structure and function of neuronal networks. Demyelinating diseases are some of the known adverse effects of the mRNA injections. In the hippocampus, the activation of microglia was associated with inhibited neurogenesis, which could explain impaired memory formation in patients. The activation of microglia appeared to be mediated by persistently elevated levels of a molecule called C-C motif chemokine 11 (CCL11). CCL11 has been associated with ageing and with inhibition of neurogenesis [177,178], as well as allergies and the recruitment of eosinophils [179]. 

Fernández-Castañeda et al. [180] investigated the effects of mild respiratory SARS-CoV-2 infection in a mouse model. They detected changes in neuroinflammatory cytokines and chemokines, including the protein CCL11 in the cerebrospinal fluid and serum over a period of 7 weeks after initiation of infection. They also observed changes specific to the brain regions of the subcortical white matter, with microglia activation and subsequent loss of oligodendrocytes, oligodendrocyte-precursor cells, and myelin.

Other authors have found CCL11 protein increases the proportion of CD4 + CD25 + Foxp3+ Treg cells, the expression of CCR3 and Foxp3, and the release of IL2 and TGFβ1 in non-tumour-associated CD4+ T cells via the STAT5 signalling pathway [60]. Regulatory T-cells are immunosuppressive and shift an immune response towards immune tolerance. This concurs with a German group [149] who showed that vaccination with mRNA-LNP complexes causes a general shift in antibodies from inflammatory IgG1 and IgG3 to IgG4, which is associated with Treg cells and immune tolerance and occurs after the second vaccination. The proportion of spike-specific IgG antibodies that were IgG4 rose from 0.04% shortly after the second dose to 19.27% late after the third vaccine dose. Demonstrative of the tolerance effect, a large Cleveland Clinic study of staff has found increasing IgG4 with subsequent booster doses correlates with increased susceptibility to SARS-CoV-2 infection [181].

Chemokines like the eotaxin CCL11 (eotaxin-1) are produced locally from epithelial, mesenchymal, and endothelial cells and are crucial for directing migration and priming of eosinophils or mediator secretion once they reach the airways [182,183]. Eosinophils secrete a range of proinflammatory granule basic proteins that include major basic protein, eosinophilic cationic protein, eosinophil-derived neurotoxin, and eosinophil peroxidase [184].

Another study looked at the toxin-like domain of the RBG on S1, which binds to α7 nAChR, increasing levels of IL-1b and TNF*α* in the brain and impaired episodic memory in mice [178]. As discussed above, the blocking of this receptor with the spike protein could cause very high levels of inflammation since it regulates pro-inflammatory cytokine production. 

The nAChR is highly expressed in the hippocampus, cortex and several limbic regions, and is involved in cognition, sensory information processing, attention, working memory, and reward pathways. Reduction in α7 in the brain, particularly in the hippocampus, has been reported in Alzheimer’s disease patients. In addition to binding the α7 nACHR in a manner similar to a neurotoxin, the spike protein has been demonstrated to be amyloidogenic [185]. It is known that amyloid β (Aβ) peptides of Alzheimer’s disease bind to the nAChRs with picomolar affinity, and that snake α-neurotoxins competitively inhibit this [186]. Amyloid has long been known to bind to nAChR receptors, as has spike protein. Computerised in silico modelling of the binding mechanism to amyloid demonstrates similarity to that of snake venom and thus it has been proposed that the interaction with AChR enables the conformational change of amyloid such that it then blocks channel opening and similar to snake venom, at low concentrations initially activates, but then slows and blocks AChR channel function. Low concentrations (picomolar) of soluble Aβ peptides in the brain of healthy people play physiological roles, whereas in Alzheimer’s disease, concentrations increase to the nanomole range and trigger the formation of insoluble plaques, a major neuropathologic hallmark of Alzheimer’s [187]. 

#### 7.3.2. Prion Formation and Neurodegenerative Effects

Neurodegenerative diseases such as Alzheimer’s, Parkinson’s disease, and amyotrophic lateral sclerosis (ALS) are all associated with misfolded proteins that accumulate in plaques and Lewy bodies. These proteins, which are termed amyloidogenic, have also been labelled as “prion-like”. Prion-like C-terminal domain of TDP-43 and α-synuclein interact synergistically to generate neurotoxic hybrid fibrils [188]. Thus at least two mechanisms exist by which spike protein, via α7 nAChR, may contribute to neurodegenerative disorders: direct inhibition and secondary amyloidogenic inhibition.

The SARS-CoV-2 spike protein receptor binding domain has prion-like properties, is the only coronavirus with such properties, and has enhanced virion binding affinity to the ACE-2 receptor and thus increased human infectivity and transmissibility [189]. The full spike protein with intact receptor binding domain (RBD) S1 subunit, if it crosses the BBB, therefore has prion-like properties that warrant further research into possible pathogenic effects.

Additionally, the mRNA or adenovectorDNA vaccines include protein sequences that can induce TDP-43 and FUS (proteins involved in RNA/DNA binding and RNA regulation) to aggregate into prion configuration. This could potentially lead to neurodegenerative conditions, such as Alzheimer’s disease [190,191]. The connection with neurodegenerative disease is the ability of the spike protein to interact with heparin-binding, amyloid-forming proteins [192]. Though speculative, these considerations are supported by a case report of prion disease due to vaccination [193]. In an in vitro experiment, spike protein was proteolyzed into smaller segments by neutrophil elastase, some of which exhibited amyloidogenic properties [185]. 

Certain primary sequences in sialoglycoproteins in neurons in the brain, enable these proteins to adopt a range of alternative structures capable of conformational self-replication via templating copies of the same protein. This conversion into what is termed prions typically radically alters the protein function, often becoming transmissible [194]. Prions thus consist of the misfolded, amyloidogenic isoform of prion protein.

Prion diseases, such as Creutzfeldt-Jakob disease (CJD) are fatal neurodegenerative disorders caused as a result of vacuolation and spongiform neuropathologic changes with rapid neurodegeneration and activation of astrocytes and microglia [195]. Neuronal accumulation of misfolded proteins is involved in the pathogenesis of other neurodegenerative disorders, including Alzheimer’s disease and Parkinson’s disease [196,197]. Infectious prion diseases may also induce nonspecific neurocognitive effects [198].

CJD cases have also been documented post-COVID-19 vaccination; one as early as 5 days post-vaccine [199] and another dying at 6 months [200]. In Australia dementia deaths for January–February 2022 were increased 27.2% above the 2017–2021 baseline (which included the first COVID-19 wave) for the same months, with an ongoing increased dementia mortality rate since then [201,202].

Neurological symptoms are commonly noted post-COVID-19, in ‘long Covid’ and post mRNA vaccination, raising the possibility of prion involvement.

Potential mechanisms by which mRNA COVID-19 vaccination could produce prions and trigger neurodegenerative processes include: RNA binding proteins like TAR DNA binding protein (TDP-43) and Fused in Sarcoma (FUS) can be activated to form disease-causing prions; TDP-43 dimers bind UG-rich RNA or TG-rich DNA and are resistant to degradation [203] and binding to these RNA sequences when the proteins are cytoplasmic may cause misfolding leading to prion formation [204]. It is, therefore, concerning that the Pfizer vaccine uses a unique RNA nucleoside 1-methyl-3’-pseudouridylyl (Ψ) and that multiple uracil motifs have been found in the vaccine mRNA [191].

In addition to uracil sequences in the mRNA which may bind proteins and precipitate misfolding, prion-like domains in the RBD of the S1 subunit of the spike protein have been identified in silico. SARS-CoV-2 is the only coronavirus with such a domain, conferring a 10- to 20-fold higher affinity for ACE-2 receptor compared to SARS-CoV-1 in addition to its prion potential [189].

Further, spike protein RBD has several heparin-binding sites that can interact with heparin and heparin-binding amyloid-forming proteins, suggesting that this peptide is prone to act as functional amyloid and form toxic aggregates [205]. The S1 protein has been demonstrated to bind stably to aggregation-prone proteins Aβ, α-synuclein, tau, prions, and TDP-43 and thus could initiate aggregation of these proteins and subsequent neurodegeneration [192].

Researchers also identified a ‘glycine zipper’ motif within the S1 subunit linked to susceptibility to misfolding and thus prion formation. It is characterised by a pattern of two glycine residues spaced by three intervening amino acids, represented as GxxxG. The GxxxG motif is a common feature of transmembrane proteins, and the glycines play an essential role in cross-linking α-helices in the protein [206]. Prion proteins become toxic when the α-helices misfold as β-sheets, and the protein is then impaired in its ability to enter the membrane [207]. Amyloid-β precursor protein (APP) has four GxxxG motifs: the glycine plays a central role in the misfolding of amyloid-β linked to Alzheimer’s disease [208]. The SARS-CoV-2 spike protein is a transmembrane protein that contains five GxxxG motifs in its sequence (see uniprot.org/uniprot/P0DTC2), one within the RBD and thus it is plausible that it could behave as a prion [209].

Another proposed mechanism is the spontaneous induction of prions and prion-like proteins via the effects of Reactive Oxygen Species (ROS). Excess ROS formation and presumed compromised mitochondrial function with cognitive dysfunction is a feature of both acute severe COVID-19, long COVID [198] and spikeopathy. Under situations of stress, TDP-43, FUS, and other RNA-binding proteins translocate from the nucleus to the cytoplasm and associate with stress granules [210,211]. When the stress dissipates, the stress granules disaggregate, and the RNA-binding proteins return to the nucleus. Enhanced environmental stress with excess ROS (for example, exposure to toxins, traumatic injury, viral infection) could cause loss of normative proteosome functioning and restoration of normal conformation, increasing the likelihood for RNA-binding proteins to inappropriately aggregate [212,213]. 

Similar is the effect of neuroinflammation, particularly astrocyte activation. Animal studies demonstrate the accelerated transition from pre-clinical to clinical stages of prion disease in settings of co-infection, with neuroinflammation, elevated pro-inflammatory cytokines, and enhanced activation of A1 reactive astrocytes [214]. TNF and C1q from activated microglia further activate A1 astrocytes [215] that are thought to be neurotoxic by mediating neuronal damage and serving as foci for prion propagation [216]. Non-neutralising antibodies after vaccination against spike protein peptides in mice have also been demonstrated to activate glial cells and astrocytes [217], consistent with this proposed mechanism of activated astrocytes, prion formation and cognitive dysfunction.

Seneff and colleagues, in an extensive narrative review of potential pathophysiological mechanisms of the spike protein in neurodegenerative diseases, describe “the spike protein’s contributions, via its prion-like properties, to neuroinflammation and neurodegenerative diseases; to clotting disorders within the vasculature; to further disease risk due to suppressed prion protein regulation in the context of widely prevalent insulin resistance” and “explain why these prion-like characteristics are more relevant to vaccine-related mRNA-induced spike proteins than natural infection with SARS-CoV-2” [29] (abstract p.1). Key findings they reviewed included:

- Spike-induced endotheliitis disturbs the blood-brain barrier and exacerbates Alzheimer’s disease via the interaction of the spike protein with amyloid β or hyperphosphorylated tau [164].
- Studies have shown that autoantibodies in the globular C-terminal domain can cause an aggressive form of Creutzfeldt Jakob Disease (CJD) by interfering with the transport of the prion protein into the endoplasmic reticulum [218].
- The spike protein itself, which is also an RNA-binding protein, may facilitate the reverse transcription of spike protein mRNA into DNA, mediated by LINE-1. Neurons actively express LINE-1 in association with neurodegenerative diseases [219,220].
- Cells that take up mRNA from the lipid nanoparticles in mRNA vaccines package up some of the mRNAs, together with the ionizable cationic lipids, into small lipid particles released as exosomes that can be shipped around the body [59,221]. For example, an immune cell in the spleen could ship intact mRNA code for the spike protein to the brain along the vagus nerve, whereupon a neuron or microglial cell could begin to synthesise spike protein.
- Micro RNA (miRNA) are small bits of active RNA code, capable of active control of cell function, including embryogenesis and apoptosis. miR-146a is found in exosomes released by immune cells and is on the list of miRNAs whose expression levels are altered in association with COVID-19 [222]. Exosomes that reach the brain stem deliver not only spike protein but potentially also intact mRNA and miRNA molecules, including miR-146a which is associated with both viral infection and prion diseases in the brain [223,224].
- Spike protein itself induces sharp TNF-α upregulation and causes cognitive issues which could indicate that it upregulates prion protein (PrP) expression in the brain. An increase in prion glycoprotein (PrP^C^) numbers can lead to prion conformation misfolding and generate prions and prion-related diseases [225,226].
- Spike protein has been shown to induce senescence in transfected cells [227]. Further, it has been proposed that the mRNA COVID-19 vaccines can induce premature senescence via syncytia formation in exposed immune cells, due primarily to their lipid content (ionizable lipids, cholesterol and the phospholipid 1,2-distearoyl-sn-glycero-3-phosphocholine (DSPC)) [228]. In vitro molecular studies show that macromolecular crowding can facilitate the conversion of native PrP into the neurotoxic soluble β oligomer configuration [229].

#### 7.3.3. Dysautonomia

Another key feature of COVID-19 infection or vaccination is dysautonomia (DSN), a neurological disorder of autonomic nervous system (ANS) function, with widespread effects on the heart, bladder, sweat glands, pupils, intestines, and other autonomic systems. Both the sympathetic (SNS) and parasympathetic nervous system (PSNS) are affected, with the potential for a sympathetic storm and abnormal autonomic responses that include excess sweating, exercise intolerance, insomnia, resting tachycardia, postural hypotension, fatigue, urinary and bowel dysfunction. The neuroinvasive nature of SARS-CoV-2 results in neurological complications such as DSN [96] and suggests either direct autonomic neuronal injury or indirect immune-mediated mechanisms, as would occur with α7 nAChR inhibition. Inhibition of nAChR by SARS-CoV-2 may lead to the inhibition of PSNS and exaggeration of SNS with subsequent progression of cytokine storm [94]. 

Another neurological dysfunction related to COVID-19 is anosmia, a common symptom of COVID-19 and also prodromal for Parkinson’s disease. The olfactory bulb has a rich network of nAChRs, and α7 nAChRs may also be expressed on the olfactory axon terminals. This may facilitate CNS infection through anterograde transport along the olfactory nerve. Anosmia may thus represent another sign of dysfunction of the nicotinic cholinergic system in COVID-19 [32].

These potential neuropathological effects are grave concerns, given the experimental nature of mRNA vaccines, the pathogenicity of the SARS-CoV-2 spike protein, and the ability of the lipid-nanoparticle carrier matrix to traverse the BBB.

### 7.4. Carcinogenic Effects

To date, there is no conclusive evidence to link mRNA injections to cancer. There is anecdotal evidence from newspapers and doctors around the world who report reactivation of cancers after years of remission. These have been termed so-called ‘turbo-cancers’, rapidly progressive to advanced stages or death. An eminent oncologist who has researched the anti-cancer potential of vaccines [230], Prof Angus Dalgleish, has drawn severe criticism for claims that this occurs post-COVID-19 vaccines. Dalgleish wrote a letter to the chief editor of the *BMJ* which he has made an open letter [231]. 

It is too early to make a definitive assessment; however, we can look at the spike protein and its potential to cause cancer or impair the immune system to the point that it cannot fight cancer effectively. In Dalgleish’s letter, he noted the preponderance of these post-COVID-19 vaccine reports were “melanoma or B-cell based cancers, which are very susceptible to immune control”.

The immune response involves the activation of a highly complex network of activator and inhibitor pathways. Immune defence coexists with the maintenance of self-tolerance and the balance between these processes is crucial. Immune checkpoints play an important role in network control. One important inhibitory checkpoint receptor is programmed cell death protein 1 (PD-1, CD279) which is typically found on T-cells, mature B-cells and other immune cells [232]. Its ligands, programmed death ligand 1 (PD-L1) and PD-L2, are regularly expressed on antigen-presenting cells like dendritic cells and macrophages; upregulation of PD-L1 is observed after activation of monocytes and granulocytes [233,234]. 

Of concern in this context, Loacker et al. [235] show that the PD-L1 expression of peripheral granulocytes and monocytes of vaccinated individuals is significantly higher than expression in the non-vaccinated. Diskin et al. [236] found that T-cell expression of PD-L1 in cancer was regulated by tumour antigen and sterile inflammatory cues. PD-L1+ T cells exerted tumour-promoting tolerance [232] via (1) binding of PD-L1 induced STAT3 (signal transducer and activator of transcription protein 3) -dependent ‘back-signalling’ in CD4+ T-cells, which prevented activation [233]; (2) PD-L1+ T-cells restraining effector T-cells and accelerating tumorigenesis, even in the absence of endogenous PD-L1 and (3) PD-L1+ T-cells engaging PD-1+ macrophages [234], inducing an alternative M2-like program, which had crippling effects on adaptive antitumor immunity. Collectively, they demonstrate that PD-L1+ T-cells drive tolerance on tumour immunity.

Furthermore, Singh and Singh [87] demonstrated in vitro interaction between the S2 subunit of spike protein with tumour suppressor proteins P53 and BRCA1 and BRCA2. Apart from direct interaction, transfection of cells with mRNA code for spike protein results in the generation of exosomes that contain miRNA (miR-148 and miR-590), which suppress interferon regulatory factor 9 (IRF9) production and activate pro-inflammatory gene transcripts [237]. 

There is evidence that Covid19 vaccination impairs the type I interferon signalling [238], which is vital for a healthy immune system. Interferon is involved in the inhibition of tumour cells and regulation of protein synthesis in immune cells, and its impairment is linked to cancer and viral diseases. Ongoing inhibition of IRF9 will suppress TNF-related apoptosis-inducing ligand (TRAIL) and all its regulatory and apoptotic effects. IRF9 suppression is also expected to impair the cancer-protective effects of BRCA2 and has been found to promote a powerful immunoinflammatory response associated with lethal neurological disease [239]. Deficiencies of IRF9 lead to a significantly greater risk of severe COVID-19 illness [240] and impair cancer-protective effects of BRCA2 gene activity.

Associated cancers include breast, fallopian tube, ovarian cancer for women, prostate and breast cancer for men, and acute myeloid leukemia in children. Liu et al. [241] showed that the mRNA injections also suppress IRF7 and STAT2 (signal transducer and activator of transcription protein 2) which can be expected to interfere with the anti-tumour effects of breast susceptibility gene 1 (BRCA1). BRCA1-associated cancers are breast, uterine and ovarian cancer in women, prostate and breast cancer in men, a moderately increased risk for pancreatic cancer in both and acute myeloid leukemia in children [242]. Reduced BRCA1 expression is linked to both cancer and neurodegeneration.

### 7.5. Biopsy and Autopsy Evidence of Spikeopathy

**Figure 7:**
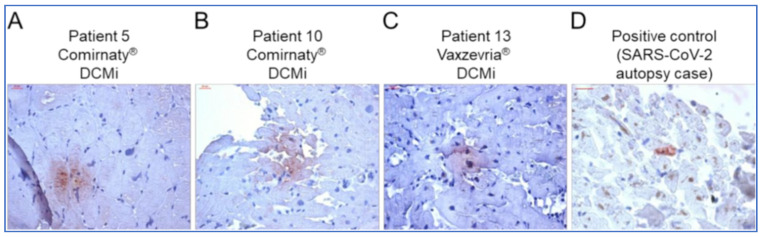
A. Evidence of SARS-CoV-2 spike protein in cardiac tissue after COVID-19 vaccination. (**A**–**C**) Representative immunohistochemical stainings of SARS-CoV-2 spike protein in EMBs from patients diagnosed with DCMi after receiving Comirnaty^®^ (panel (**A,B**), patients 5 and 10) or Vaxzevria^®^ (panel (**C**), patient 13). (**D**) SARS-CoV-2-positive cardiac tissue served as positive control. Magnification 400×. Scale bars 20 μm. Reprinted with permission from Ref. [103]. Copyright 2022 MDPI.

A German multisite case series of 15 suspected post-COVID-19 vaccine myocarditis cases (eight Pfizer, two AstraZeneca, two Johnson & Johnson) performed a comprehensive immunohistopathological examination of endomyocardial biopsies (EMBs). Immunological tests for previous SARS-CoV-2 or other viruses associated with myocardial inflammation were negative. All but one of the patients exhibited inflammatory biomarkers and diagnoses of inflammatory cardiomyopathy, active myocarditis, and severe giant cell myocarditis. Nine of the 14 patients stained positive for intramyocardial spike protein. When lymphocyte infiltrates were examined, the preponderance of CD4^+^, versus CD8^+^ T-cells led the authors to conclude an autoimmune reaction was the basis of the pathology [103]. Similarly, the relative frequency of HLA-D4-activated lymphocytes and MAC-1+ macrophages was taken to support this conclusion. Figure 7 represents some of the tissue stainings of spike proteins [103].

An autopsy case of a 76-year-old man revealed COVID-19 vaccine spike proteins were the cause of death [167]. Clinically this subject had cardiovascular adverse effects from the day of his first vaccination (AstraZeneca) in May 2021, and neurological and psychiatric changes after his second vaccination (Pfizer) in July 2021, then collapsed and died 3 weeks after his booster (Pfizer) in December 2021. Acute and chronic inflammatory and cellular degeneration changes affected his brain and heart. Immunohistochemical stains showed spike protein in blood vessel walls, in glial cells in the brain, and in “cardiac endothelial cells that showed lymphocytic myocarditis” [167] (p. 8). Additionally, “immunohistochemical staining did not detect the SARS-CoV-2 nucleocapsid protein”, such that the author concluded: “the presence of spike protein must be ascribed to vaccination rather than to viral infection” [167] (abstract).

**Figure 8:**
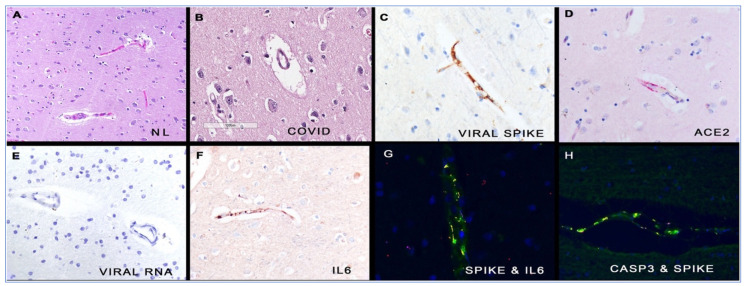
Histologic and molecular correlates of COVID-19 in human brains. Panel (**A**) shows the microvessels in normal brain. In comparison, many of the capillaries in COVID-19 brain tissues show marked perivascular oedema (panel (**B**)). Serial section analyses of the COVID-19 brain shows that the endothelial cells of the microvessels contained the spike glycoprotein (panel (**C**)), the ACE2 receptor (panel (**D**)) and IL 6 (panel (**F**)), but not viral RNA (panel (**E**)). The fluorescent yellow signal marks co-localisation of the spike protein with IL6 (panel (**G**)) and caspase 3 (panel (**H**)), respectively, in these endothelial cells. Each magnification is 800× with DAB (brown) signal (panels (**C**–**F**)) or Fast Red (red) (panel **D**). (For interpretation of the references to colour in this figure legend, the reader is referred to the web version of this article.). Reprinted from Annals of Diagnostic Pathology, Vol. 51, Nuovo GJ, Magro C, Shaffer T. et al., Endothelial cell damage is the central part of COVID-19 and a mouse model induced by injection of the S1 subunit of the spike protein. Figure 1, 151682, Reprinted with permission from Ref. [243]. Copyright (2020) Elsevier.

A case series of 13 brain autopsies in the USA found the S1 subunit (inclusive of the receptor binding domain RBD) of the spike protein caused degeneration of neurovascular endothelial cells with endotheliitis, cytokine release, and neural capillary damage. Endothelial cells contained spike glycoprotein but not viral RNA, thus confirming free spike protein S1 subunit/RBD to be the main pathogenic agent in these cases of COVID-19 disease, as shown in Figure 8 [243].

The same research group found similar findings in a case series of 11 autopsies of the hearts from patients who had died from COVID-19 [244]. They noted the following details, with the strong implication that the expression of foreign spike antigen was pathologically significant:

“*Cardiac disease in fatal COVID-19 is associated with viral spike protein, but not the infectious virus. The viral spike protein is endocytosed in interstitial macrophages and induces myocarditis. The histological findings show perivascular oedema, endothelial cell damage, and microthrombi*”.

[244] (highlights, p. 1)

The findings of this US research group confirm spikeopathy as the pathogenic mechanism for neurovascular and cardiac pathology of COVID-19. Therefore, widely biodistributed genes coding for prolonged spike protein production in the brain and heart via the COVID-19 vaccines would likely follow the same mechanisms of action.

A case report of an autopsy in a previously healthy 22-year-old male military recruit in South Korea revealed extensive myocarditis five days after Pfizer mRNA COVID-19 vaccination. The authors stressed the importance of conducting histopathological investigations during autopsies. They noted a marked deficiency of such investigations in the academic literature during the COVID-19 pandemic [245].

German pathologists have performed a series of autopsies on the bodies of people who died shortly after vaccination and had not previously had COVID-19 illness. The chief pathologist in Heidelberg, Dr Schirmacher and colleagues, autopsied 35 people who died at home within two weeks of COVID-19 mRNA vaccinations. Ten were found to have died from pre-existing illnesses. Of the remaining 25 unexpected deaths, five were found to have died from myocarditis, with lymphocytic inflammatory infiltrates of the myocardium and presumed associated arrhythmia in the absence of any other significant cardiovascular pathology. All five died within one week, one within 12 h of vaccination. One case had a similar inflammatory infiltrate at the deltoid muscle injection site [246]. 

**Figure 9:**
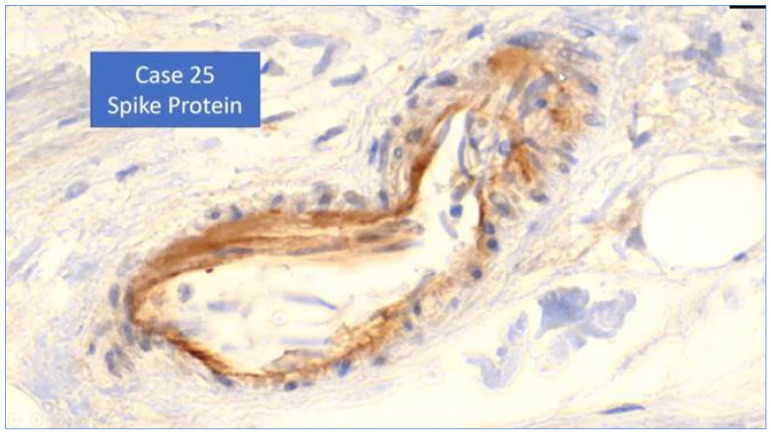
Spike protein in blood vessel wall from Burkhardt (2022a) [247].

A separate group of German pathologists, led by Prof Arne Burkhardt and colleague Prof Walter Lang, presented histopathological findings in a case series of 25 autopsies post-Pfizer COVID-19 mRNA vaccination. These findings included vaccine-produced spike proteins in lesions in blood vessels as well as in inflammatory infiltrates in myocarditis. Figure 9 is from a conference PowerPoint presentation in German describing immunohistopathological-stained mRNA-produced spike proteins [247], and shows the stained (in brown) spike proteins infiltrating endothelial cells of a blood vessel wall:

The translation from German accompanying this slide, in the words of Prof Arne Burkhardt, is:

“*Yes, that is the finding that we have now been able to collect using special methods. This means that we are actually certain that in this case, we can still detect this toxin in the vessel walls 122 days after the vaccination. It is also clear that this is the causal factor for this damage*.

*What’s not clear to me at the moment is whether it’s just deposited there or whether these cells actually produce the spike protein, as they are told to do by the mRNA, so to speak*.”

**Figure 10:**
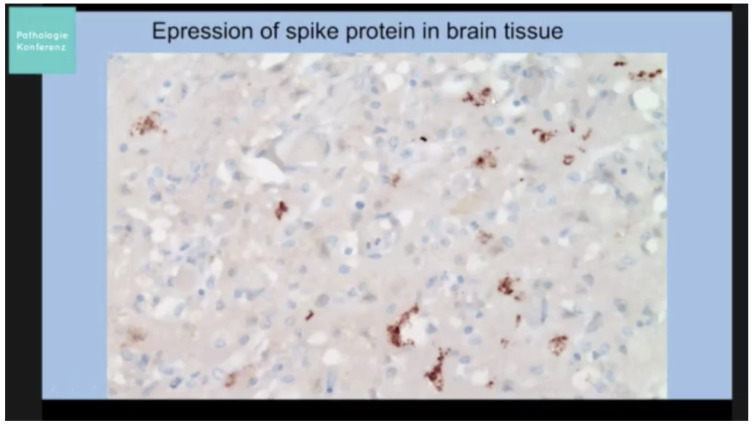
Spike protein in brain tissue from Burkhardt (2022b) [248].

Figure 10 is from the same autopsy case series by Burkhardt and colleagues, showing that either the spike protein has traversed the blood-brain barrier, or that the lipid-nanoparticle-mRNA complex has done so and caused the transcription of spike proteins in brain tissue [248].

This presence of spike proteins in pathological tissue that has contributed to patients’ demise could be described as analogous to finding a ‘smoking gun’ at the scene of a crime, only the ‘shot’ was delivered in some cases months earlier. Clearly, more autopsies using such staining methodologies and related research are required.

A recent case series of three autopsies in patients who demised from VITT from adenovectorDNA vaccines of AstraZeneca or Janssen/Johnson & Johnson used histopathogical staining of the cerebral venous sinus thrombi [7]. The authors report:

“*Endothelial cells adjacent to the thrombus were largely destroyed. Markers of neutrophil extracellular trap and complement activation were present at the border and within the cerebral vein thrombi. SARS-CoV-2 spike protein was detected within the thrombus and in the adjacent vessel wall*”.

[7] (abstract)

This finding of spike proteins at the site of the cerebral venous thrombi from viral-vectorDNA COVID-19 vaccines, and the presence of NETosis as described above, provides yet further evidence for spikeopathy from gene-based vaccines.

## 8. Discussion

We began this paper by quoting the Australian health regulator, the TGA,’s response to an Australian Senator’s question as to the risks of gene-based vaccines that induce human cells to manufacture the SARS-CoV-2 spike protein. The response was that the spike protein was not a pathogen. We have presented significant evidence that the spike protein is pathogenic. This applies when it is part of the virus, when it is free but of viral origin, and when it is produced in ribosomes by the mRNA of the mRNA and adenovectorDNA COVID-19 vaccines. Pathophysiological mechanisms of action of the spike protein continue to be elucidated. 

We established that the spike protein causes damage by binding to the ACE-2 receptor and thereby downregulating the receptor, damaging vascular endothelial cells. The spike protein has a toxin-like binding domain, binding to α7 nAChR in the central nervous system and immune system, thereby, interfering with nAChR functions, such as the function to reduce inflammation and proinflammatory cytokines, such as IL-6. The link to neurodegenerative diseases is also through the ability of the spike protein to interact with the heparin-binding amyloid-forming proteins initiating aggregation of brain proteins.

The persistence of the spike protein causes persistent inflammation (chronic inflammation), which potentially eventually shifts the immune system into immune tolerance (IgG4). A particular effect for women and pregnancy is the binding of the spike protein to oestrogen receptor alpha, which interferes with oestrogen messaging. 

The spike protein is cytotoxic inside the cells by interaction with cancer suppressor genes and causing mitochondrial damage. Spike proteins expressed on the surface of cells leads to cytopathic autoimmune response. 

Free spike protein binds to ACE-2 on other cells of organs and blood. In the blood the spike protein influences platelets to release coagulation factors, secrete inflammatory factors and forms leukocyte-platelet aggregates. The spike protein binds fibrinogen, inducing blood clots.

There is also problematic homology of the spike protein to key proteins in the adaptive immune system leading to autoimmunity if vaccinated with the mRNA producing spike protein.

Pharmacokinetic factors contribute to the pathophysiology. As mentioned the Pfizer biodistribution study (where 75% of lipid-nanoparticle carrier molecules left the deltoid for all organs within 48 h) for the Japanese PMDA was known to the Australian TGA before the provisional authorisation of the mRNA COVID-19 vaccines for the Australian population [5]. Because they cause replication of the spike protein in many organs, the gene-based vaccines act as synthetic viruses.

The lipid-nanoparticle carrier of the mRNA and the associated PEG that makes the mRNA-LNP complex more stable and resistant to degradation, have their own toxic effects; the lipid-nanoparticles primarily via pro-inflammatory effects and PEG by anaphylaxis in susceptible individuals.

Röltgen et al. [53] found the N1-methylpseudouridine stabilised mRNA in the COVID-19 vaccines produces spike proteins for at least 60 days. Other cited research on retroposition of the genetic code [249], suggests the possibility that such production of a foreign pathogenic protein could potentially be lifelong or even transgenerational. 

A large body of emerging research shows that the spike protein itself, in particular the S1 subunit, is pathogenic and causes inflammation and other pathology seen in severe acute COVID-19, likely in long COVID, and in mRNA and adenovectorDNA COVID-19 vaccine injuries. The word ‘spikeopathy’ was coined by French researcher Henrion-Caude [98] at a conference and given the varied and substantial pathological effects of the SARS-CoV-2 spike protein, we suggest the use of the term will have heuristic value.

Spikeopathy exerts its effects, as summarised by Cosentino and Marino [86] through ACE-2 binding related platelet aggregation, thrombosis and inflammation; disruption of CD147 transmembrane glycoproteins that interfere with cardiac pericyte and erythrocyte function; binding to TLR2 and TLR4 igniting inflammatory cascades; binding to ER alpha possibly responsible for menstrual irregularities and increasing cancer risk via interactions with p53BP1 and BRCA1. Other research shows further spikeo-pathological effects via ACE-2-induced inflammatory cytokine production, phosphorylation of MEK, and downregulation of eNOS, impairing endothelial cell function.

Particularly novel effects of the spike protein involve the derangement of the nicotinic cholinergic system via inhibition of α7 nAChR, leading to impaired anti-inflammatory biochemical pathways in many cells and organ systems, as well as impaired parasympathetic vagal tone.

COVID-19 mRNA and adenovectorDNA vaccine injuries overlap with severe acute COVID-19 illness and long COVID, but are more varied, given the wider biodistribution and prolonged production of the spike protein. Myopericarditis is recognised but often has been downplayed as mild and rare, yet the evidence for relatively common subclinical COVID-19 vaccine-related myopericarditis [113,115] and autopsy evidence [246,247,248] suggests a role in sudden deaths in relatively young and fit people [116,117]. Spike proteins also have mechanisms for increasing thrombosis via ACE-2-related inflammation, disturbance of the angiotensin system [119], direct binding with ACE-2 receptors on platelets [1], disruption of antithrombin [122], delaying fibrinolysis [123] (preprint), and reducing erythrocyte electrostatic repulsion leading to haemagglutination [124].

New onset autoimmune diseases post COVID-19 vaccination might relate to the homology of the spike protein, and in the viral disease including other SARS-CoV-2 proteins, with human proteins [5,138].

The mRNA-LNP complex crosses the BBB and neurological disorders are highly reported to pharmacovigilance databases following COVID-19 vaccines. A number of mechanisms of spikeopathy are being elucidated as underlying disorders involving: BBB permeability [128]; mitochondrial impairment [168]; dysregulation of brain vascular pericytes [169]; TLR4 mediated neuroinflammation [170]; hippocampal cell death [171]; dysregulation of complement and coagulation cascades and neutrophils causing coagulopathies [173] (preprint); neuroinflammation and demyelination via microglial dysregulation [174,177,180]; increased expression of α-Syn involved with neurodegenerative disease [175]; elevated levels of C-C motif chemokine 11 associated with ageing and subsequent loss of neural cells and myelin; binding to the α7 nicotinic acetylcholine receptor (nAChR), increasing levels of IL-1b and TNF*α* in the brain causing high levels of inflammation [172,177]; the S1 subunit is amyloidogenic [185]; dysautonomia [96], by either direct neuronal injury or indirect immune-mediated mechanisms, e.g., α7 nAChR inhibition; anosmia caused by both vaccine and disease [44], also prodromal to Parkinson’s disease. 

Furthermore, autoantibodies in the globular C-terminal domain can cause Creutzfeldt Jakob Disease (CJD) [218], miR-146a is altered in association with COVID-19 [222] and associated with both viral infection and prion diseases in the brain, and S1 has been shown to induce senescence in transfected cells. 

The amount of possible mechanisms of spike-mediated damage in the brain is matched in real life by the prevalence of neurological and neurodegenerative adverse effects and urgently requires further research.

Cancer—although not definitely proven to be caused by the vaccines—seems to follow vaccination closely and we have reviewed possible causes in the form of spike protein interactions with transcription factors and cancer suppressor genes. 

The vaccine was meant to protect the over age 60 with the greatest risk of mortality from COVID-19 [10], yet a risk analysis by Dopp and Seneff (2022) [250] showed that the likelihood of dying from the injection is only 0.13% lower than the risk of dying from the infection in those aged over 80 years. 

Furthermore, natural aging is accompanied by changes in the immune system that compromise the ability to effectively respond to new antigens. Similar to age-stratified responses to viruses, this means vaccines become less effective in inducing immunity in the elderly resulting in a reduced ability to fight novel infections [251]. Two-dose COVID-19 mRNA vaccination conferred limited adaptive immune response among aged mice, making them susceptible to SARS-CoV-2 infection [252]. The risk of severe disease among US veterans after vaccination remained associated with age according to a study by Vo et al., (2022) [253]. This risk of breakthrough infections was also higher if immunocompromising conditions were present.

Finally, we reviewed the best autopsy case series currently available, performed in Germany, that establish the connections between spikeopathy and multiple organ failures, neuropathies and death.

## 9. Conclusions

In this narrative review, we have established the role of the SARS-CoV-2 spike protein, especially the S1 subunit, as pathogenic. It is also now apparent that widely biodistributed spike proteins, produced by mRNA and adenovectorDNA gene codes, induce a wide variety of diseases. The underlying pathophysiological and biochemical mechanisms are being elucidated. The lipid-nanoparticle carriers for the mRNA and Novavax vaccines have pathological pro-inflammatory properties as well. The whole premise of gene-based vaccines producing foreign antigens in human tissues is fraught with risks for autoimmune and inflammatory disorders, especially when the distribution is not highly localised. 

The clinical implications that follow are that clinicians in all fields of Medicine need to be mindful of the varied possible presentations of COVID-19 vaccine-related illness, both acute and chronic, and the worsening of pre-existing conditions. We also advocate for the suspension of gene-based COVID-19 vaccines and lipid-nanoparticle carrier matrices, and other vaccines based on mRNA or viral-vectorDNA technology. A safer course is to use vaccines with well-tested recombinant protein, attenuated or inactivated virus technologies, of which there are now many for vaccinating against SARS-CoV-2.

## Figures and Tables

**Table 1:** Studies demonstrating persistence of vector-based vaccine constituents and/or derivative spike protein.

Author	Constituents/Tissue Type/Assay Technique	Duration Measured
Animal		
Pfizer (Japanese MoH) 2020 [46]	Radiolabelled LNP in plasma and tissues	140 h–14 days
**Human**		
Ogata et al. (2021) [52]	Spike protein and S1 subunit (assay)	3 days
Bansal et al. (2021) [57]	Spike Protein	4 months
Fertig et al. (2022) [50]	LNPs and mRNA	15 days
Röltgen et al. (2022) [53]	mRNA and Spike Protein in ipsilateral lymph nodes; 2–7 days post dose in blood	60 days
Yamamoto et al. (2022) [58]	Spike Protein in skin	3 months
Yonker et al. (2023) [54]	Spike Protein in blood	1–19 days *in cases of myocarditis*
Castruita et al. (2023) [51]	mRNA in plasma	28 days

## Data Availability

Data used in Figure 1 was derived from the official NSW Health data in the NSW Health report’s table shown in Figure 2.
